# An Application of Multivariate Data Analysis to Photoacoustic Imaging for the Spectral Unmixing of Gold Nanorods in Biological Tissues

**DOI:** 10.3390/nano11010142

**Published:** 2021-01-08

**Authors:** Mirko Maturi, Paolo Armanetti, Luca Menichetti, Mauro Comes Franchini

**Affiliations:** 1 Department of Industrial Chemistry Toso Montanari, University of Bologna, Viale Risorgimento 4, 40136 Bologna, Italy; mauro.comesfranchini@unibo.it; 2 National Research Council (CNR), Institute of Clinical Physiology, Via Moruzzi 1, 56124 Pisa, Italy; paolo.armanetti@ifc.cnr.it (P.A.); luca.m@ifc.cnr.it (L.M.)

**Keywords:** gold nanorods, photoacoustic imaging, contrast agents, spectral unmixing, multivariate analysis

## Abstract

Gold nanorods (GNRs) showed to be a suitable contrast agent in photoacoustics (PA), and are able to provide a tunable absorption contrast against background tissue, while a detectable PA signal can be generated from highly localized and targeted areas. A crucial issue for these imaging techniques is represented by the discrimination between exogenous and endogenous contrast and the assessment of the real PA signal magnitude. The application of image resolution/unmixing methods was implemented and optimized to recover the relative magnitude spectra and distribution maps of image constituents of the biological sample based on multivariate analysis (multivariate curve resolution—alternating least squares, MCR-ALS) in the presence of GNRs with tunable absorption properties. The proposed data analysis methodology is demonstrated on real PA images from experimental animal models and ex-vivo preparations.

## 1. Introduction

Nowadays, hyperspectral photoacoustic (PA) imaging of endogenous contrasts in biological systems shows good potential as it is exploitable for the study of tumor angiogenesis and melanoma [1,2]. A relevant number of contrast agents for photoacoustic imaging (PAI) has been developed [3,4,5,6,7] and amongst them, plasmonic nanoparticles have gained considerable interest over the past few decades due to their surface plasmon resonances, relative biological stability/biocompatibility, and easy functionalization. [8,9] Indeed, gold nanorods (GNRs) represent an ideal contrast agent for PA since they can provide an enhanced optical absorption contrast against background tissue [10,11,12]. Their tunable longitudinal surface plasmon resonance (LSPR) properties allow for the preparation of tissue-specific theranostic platforms exploiting the contrast they provide in photoacoustic imaging and their photothermal properties, which can be exploited for laser ablation-based therapeutic techniques [13,14,15]. The use of hyperspectral imaging coupled with multivariate resolution analysis techniques could represent a powerful approach in studying the biological variation of the treated samples, and developing new biomedical applications. Hyperspectral images combine the spatial information of optical microscopy and the biochemical information provided by a spectroscopic technique. In the case of biological samples, contributions of many different molecules (i.e., oxy- or deoxy-hemoglobin), the variability of the biological components in vivo, and the limited PA signal amplitude are often very critical to the interpretation of the PA spectra and the multivariate resolution image. To resolve this issue, the use of image resolution/unmixing methods can aid in recovering the single component of the spectra and defining the image constituents of the biological sample. Due to the high variability of in vivo micro-environment, specifically the within-tissue composition and molecular variability, defining the association between molecular absorption and image constituents could be challenging to solve.

To extract the spectral information of biological samples, multivariate analysis techniques represent an option capable to discriminate the spectral trends of the above-mentioned sample constituents [16]. Several strategies have been reported in the literature for multivariate analysis of hyperspectral data (principal component analysis, PCA, and principal component regression, PCR), which are mostly coupled to spectroscopic imaging techniques like fluorescence microscopy, NIR, and Raman imaging [17,18,19,20,21]. Among them, multivariate curve resolution—alternating least squares (MCR-ALS, Figures S1 and S2) became a popular chemometric tool for the resolution of multiple-component responses in complex mixtures when dealing with hyperspectral imaging data [22,23,24]. Up to date, the application of multivariate analysis in the PA field has not yet been fully explored for the assessment of the different sources of endogenous contrast, although its potential for discriminating between exogenous contrast agents is relevant [25,26,27]. Recently, additional complex mathematical approaches were implemented for the study of PA imaging data (such as deep learning and neural networks) that apply non-linear models to perform spectral unmixing [28,29,30]. However, these approaches appear less versatile than linear models since they include much more detailed information about the system, such as the modelling of the propagation of light inside the tissue, and are strongly dependent on the type of tissue and the instrumental parameters selected for the experiment. 

This work aims to report the performances of MCR-ALS analysis in the field of nanoimaging exploiting I techniques, and we present its application in different data set using GNRs as imaging agents. In order to validate our approach, we selected both a test object and an experimental model.

**Figure 1:**
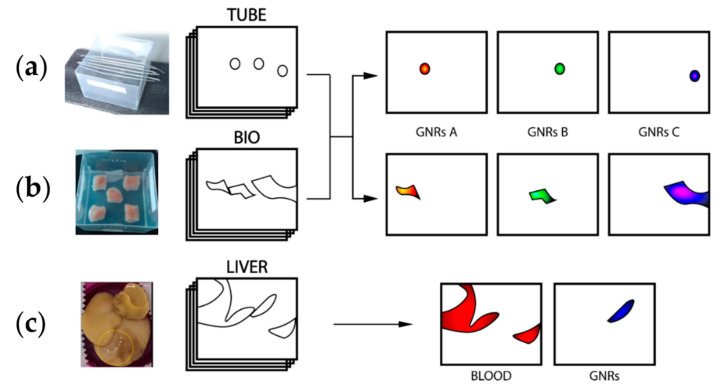
Schematic representation of the approach described in this work. Gold nanorods (GNRs) with different absorption properties underwent multispectral photoacoustic (PA) imaging in polyethylene (PE) tubes and chicken breasts generating “TUBE” and “BIO” multispectral datasets (**a**,**b**). Then, the multivariate analysis was applied to the combined datasets generating spatial distributions of the different contrast agents in the imaged section. Then, as a case study, the algorithm was applied to the multispectral PA imaging data of GNRs in tumor-bearing mice liver, the “LIVER” data set (**c**).

Briefly, MCR-ALS analysis was first applied to assess the distribution of three distinct PEGylated synthetic GNRs, with LSPR absorption peak ranging from 700 to 950 nm in a phantom with a controlled geometry (“TUBE”, Figure 1a) and in chicken breast (“BIO”, Figure 1b). After these tests, MCR-ALS was applied to map the regional distribution of GNRs in mouse livers with hepatocarcinoma, in which the tumors were treated with functionalized gold nanoparticles (Figure 1c) [10]. Finally, the MCR-ALS was semi-quantitatively compared with a reference PA built-in unmixing tool to evaluate their performances.

## 2. Materials and Methods

### 2.1. Synthesis of PEGylated GNRs

All chemicals were purchased from Sigma-Aldrich Co. (St. Louis, MO, USA) and used as received. Polyethylene glycol with amino, methoxy and thiol end groups (HS-PEG-NH_2_ and HS-PEG-OMe, MW ~3 kDa) was purchased from Rapp Polymere GmbH (Tübingen, Germany).

GNRs were prepared with the seed-mediated growth method with slight modifications of the procedures described by El-Sayed and Murray [31,32]. In total, three separate batches were prepared to achieve tunable NIR absorption of the contrast agents, named GNRs A, B, and C. All batches share a common procedure, but the relative amounts of each reagent vary for different GNRs batches and are reported in Supplementary Table S1. In the general procedure, hexadecyl trimethyl ammonium bromide (CTAB) and sodium oleate are dissolved in water by gentle heating. Once they are completely dissolved, the solution is allowed to equilibrate at 30 °C, then a certain amount of AgNO_3_ 400 mM is added under stirring conditions. The solution is then left unstirred for 15 min and HAuCl_4_ 100 mM is added while stirring at 700 rpm. Complete reduction of Au(III) to Au(I) happens in 90 min stirring at 700 rpm. While waiting for the reduction of gold in the growth solution, Au seeds are prepared by the traditional synthesis for CTAB-capped seeds. In total, 364 mg of CTAB are dissolved in 9.975 mL of water by gentle heating. After the solution cooled down to room temperature, 25 µL of HAuCl_4_ 0.1 M are added. Then, 600 µL of ice-cold 10 mM NaBH_4_ are rapidly injected under vigorous stirring. Seeds are formed by stirring for 2 min followed by ageing for 30 min. After complete reduction of Au(III) to Au(I) in the growth solution, HCl 37% is injected into the growth solution to regulate pH, and the solution is allowed to stir for 15 min at 400 rpm. To complete the gold reduction, ascorbic acid 78 mM is added. After vigorous stirring for 30 s (900 rpm), a defined amount of seed solution is injected and after a further 30 s of vigorous stirring, GNRs are allowed to grow overnight at 30 °C. The next day, CTAB-coated nanostructures are purified by centrifugation (100 min × 6 krpm) and redispersion in H_2_O (×3) and finally collected in 50 mL of water.

CTAB-coated GNRs underwent a ligand exchange reaction in order to remove cytotoxic CTAB and to replace it with biocompatible PEGs. To do so, a certain amount of GNRs@CTAB A, B, and C (10.3, 9.6, and 12.6 mL for GNRs A, B, and C, respectively) are separately diluted to a final volume of 30 mL with water, then 80 mg of HS-PEG-OMe (MW = 3000 Da) and 80 mg of HS-PEG-NH_2_ (MW = 3000 Da) are added to each separate GNRs type. Mixtures are sonicated for 1 h then stirred overnight to ensure ligand exchange and surface stabilization. Finally, PEGylated GNRs are purified by repeated centrifugation and washes with water (6 krpm, 40 min, 2 times) and finally collected in the appropriate amount of water that allows obtaining a final gold concentration of 0.5 mM.

### 2.2. GNRs Characterization

Gold concentration in GNR solutions was determined before and after the ligand exchange reaction by atomic absorption spectroscopy (SpectraAA 100 Varian, Agilent Technologies Inc., Santa Clara, CA, USA) using air-acetylene flame for atomization. Standard solutions at 1, 2, 5, and 10 mg L^−1^ of atomic gold were analyzed to obtain a calibration curve. GNRs were previously digested with aqua regia at room temperature and diluted with water to achieve a gold concentration that could fit in the calibration curve. 

VIS-NIR spectroscopy was performed with a Cary5000 double-beam spectrometer (Agilent Technologies Inc., Santa Clara, CA, USA) on 500 μM GNRs samples using water as a reference and scanning from 400 to 1100 nm. 

Transmission Electron Microscopy was performed with an FEI TECNAI F20 microscope (Thermo Fisher Scientific, Waltham, MA, USA) operating at 200 keV after drop-casting a perforated carbon film supported by a copper grid with an aqueous dispersion of GNRs. The preparation was then dried at 100 °C.

Dynamic light scattering measurements were performed on a Malvern Zetasizer-Nano-S (Malvern Panalytical, Malvern, UK) working with a 532 nm laser beam. ζ potential measurements were conducted in DTS1060C-Clear (Malvern Panalytical, Malvern, UK) disposable zeta cells at 25 °C.

### 2.3. Photoacoustic Imaging Setup

The PA tests were performed using the multimodal (PA-US) imaging platform VEVO Lazr 2100 (by Fujifilm Visualsonics Inc., Toronto, ON, Canada). PA data were acquired by imaging 500 μM PEGylated GNRs, with different aspect ratios (GNRs A, B, and C), loaded in small PE tubes or directly injected into a chicken breast tissue. CTAB-coated GNRs were prepared following a procedure available in the literature, and ligand exchange with thiol-bearing PEGs allowed for bio-compatibilization [33,34]. Details of the synthesis and characterization of the nanostructures are reported in the Supplementary Materials. Data related to the acquisitions in test objects (made by PE tubes filled with standard solutions) will be referred to as the “TUBE” data set, while data related to acquisitions in chicken breasts will be referred to as the “BIO” data set (Figure 1a,b).To assess the capability of the model to resolve the endogenous and exogenous PA contrast, MCR-ALS was applied to PA-imaged livers of mouse model treated with GNRs [6]. This last data set will be referred to as the LIVER data set (Figure 1c). 

### 2.4. PA in a Standard Sample (“TUBE” Data Set)

The PA tests were performed by analyzing the PA multispectral signal amplitude of GNRs at different longitudinal surface plasmon resonances (LSPR) in the optical windows of near-infrared I (NIR I), corresponding at the wavelength range between 680 and 970 nm. The GNRs were loaded in a coplanar net of polyethylene tubes inserted in a polypropylene box (PA panel I), then the PA probe was coupled to the samples by water to prevent possible artefacts (Supplementary Figure S3a). To avoid reshaping of GNRs under PA excitation, a homogeneous thin layer of biological tissue (chicken breast) was put on top of the tubes. The PA characteristics of GNRs and the surrounding biomatrix before the injection of GNRs were studied using PA multispectral analysis (Supplementary Figure S3b–d) to find their specific spectral fingerprint and, under prolonged laser stimulation at their LPRS peaks, to assess the photostability of the PA signal over time (around 40 s, corresponding at over 200 laser shots). The data set produced with these acquisitions will be referred to as the “TUBE” data set. 

### 2.5. Ex-Vivo PA Evaluation in Chicken Breast

As reported [35], a chicken breast sample injected with a standard solution of GNRs was produced (for simplicity, identified as “BIO” data set). A bolus of the order of 100 μL was prepared and injected ex-vivo in a chicken breast specimen. Then, the spectral distributions of the PA signals generated inside the chicken breast volume by laser stimulation were evaluated (Supplementary Figure S4). Images were acquired using PA multispectral analysis between 680 nm and 970 nm at 2 nm steps, generating 146 images per analyzed specimen. Since each image had a size of 503 × 647 pixels, the total data set contained 325,441 spectra for each acquisition. From the image size and the corresponding number of pixels, the pixel size was estimated to be around 36 μm.

### 2.6. Ex-Vivo PA Evaluation in Mice Liver

The PA images contained in this data set were acquired in ex-vivo samples obtained as reported previously [6]. Employed GNRs displayed a maximum absorption wavelength of 820 nm. PA images were acquired in the range 680–970 nm at 2 nm steps, generating 146 images per analyzed section. Since each image had a size of 505 × 648 pixels, the total data set contained 327,240 spectra for each acquisition.

### 2.7. Multivariate Analysis

The data post-processing was conducted on the Matlab R2018a platform (The MathWorks Inc., Natick, MA, USA), exploiting the MCR-ALS toolbox developed by Jaumot et al. in 2005 and optimized in 2015 [36,37]. An overview of the theory involving the MCR-ALS approach can be found in the Supplementary Materials. Briefly, it involves an iterative calculation that employs least-squares approaches to solve Equation (1) under the obligation to comply with some mathematical constraint, generating the optimal results for **C** and **S^T^** and minimizing the matrix of residuals, **E**.

**D** = **C**·**S^T^** + **E** (1)

where **D** is the raw data set, **C** contains the distributions profiles of the modelled chemical species, **S^T^** is their corresponding PA spectra and **E** represents the residual, the portion of data that is not included in the model. Non-negativity of both concentration profiles and spectra was set as a constraint for the multivariate analysis.

The result is formulated when convergence is achieved in two consecutive iterative cycles, with deviations of the residuals between experimental and ALS data less than 0.05%. The overall computing time required to run the algorithm on the input data set was lower than 5 min employing an Intel^®^ Core™ i7-7700 processor (Intel Corporation, Santa Clara, CA, USA) with CPU@3.60 GHz and 32 GB of RAM.

## 3. Results and Discussion

### 3.1. PEGylated Gold Nanorods

**Figure 2:**
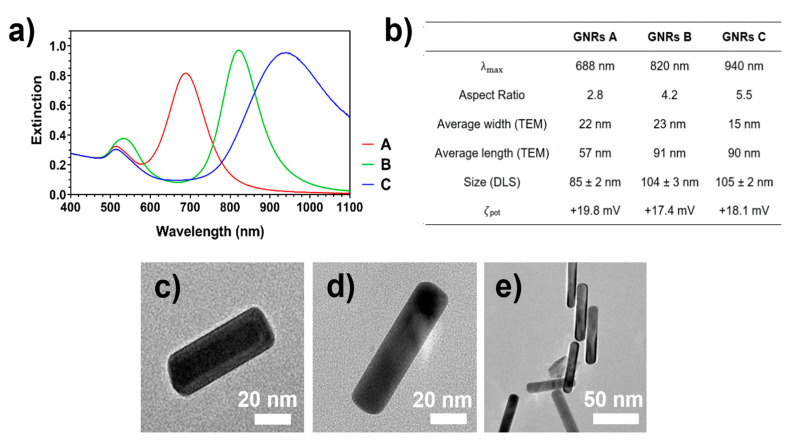
Characterization of PEGylated GNRs. (**a**) VIS-NIR extinction spectra of GNRs in water. Gold concentration was 100 μM for the three specimens. (**b**) Summary of GNRs characterization parameters. Average TEM size has been obtained by measuring *n* = 50 different GNRs for each synthetic batch. (**c**–**e**) Representative TEM images of GNRs A, B, and C, respectively.

CTAB-coated Gold Nanorods (GNRs) were synthetized according to the previously reported seed-mediated growth method. [31,32] After ligand exchange with thiolated PEGs and purification, the three samples underwent VIS-NIR extinction analysis to assess the outcome of the syntheses in terms of their optical properties. The extinction analysis revealed the presence of high-intensity LSPR bands for GNRs A, B and C centered at 688, 820, and 940 nm, respectively (Figure 2a).

By adjusting the concentrations of surfactants, silver ions, and reducing agents, the aspect ratio of the prepared GNRs was 2.8, 4.2, and 5.5 for GNRs A, B, and C, respectively. These values were obtained from the wavelength of the longitudinal surface plasmon resonance (LSPR) peak according to the formula developed by Link et al. [38] and showed herein as Equation (2):

(2) $$AR=\frac{\lambda_{max}-495.14}{53.71\cdot \varepsilon_{m}}+0.79$$

where *AR* is the aspect ratio of the GNRs sample, $\lambda_{max}$ is the wavelength corresponding to their LSPR peak and $\varepsilon_{m}$ is the refractive index of the medium (for water $\varepsilon_{m}=1.77$).

These results were also confirmed by electron microscopy (Figure 2c–e) from which aspect ratios were calculated by measuring different GNRs (*n* = 50) as 2.6, 4.0, and 6.0 for GNRs A, B, and C respectively.

The nanosystem size determined by DLS is in agreement with the size determined by TEM, with no signs of aggregation phenomena in solution. Finally, dynamic light scattering was used to determine the hydrodynamic radius of the nanosystem and surface zeta potential measurements were performed to assess the stability of GNRs due to electrostatic repulsion between different nanoparticles. Results are summarized in Figure 2b, and they are compatible with the GNRs size determined by TEM and the presence of cationic amino groups on their surface.

### 3.2. Photoacoustic Imaging Analysis

The PA analysis of the test objects confirmed the expected spectral trend of GNRs as shown in Supplementary Figure S3. The PA signal of GNRs was stable during prolonged laser illumination at the LPSR peak wavelengths, with variation coefficients and signal-to-noise ratios ranging from 3.2% to 4.2% and 24 to 31, respectively (Supplementary Tables S2 and S3). Moreover, a linear relationship between GNRs concentration and PA signal was assessed (Supplementary Figure S5).

Raw photoacoustic data were extracted from the Vevo platform as *.dicom files, containing the hypercubes for each selected projection in arbitrary units, co-registered with the US image. The raw PA signal intensity at the different recorded wavelengths, overlapped with the corresponding US trace, is available in Supplementary Videos S1–S3. 

Multivariate analysis was performed twice:

- “TUBE” and “BIO” data sets have been combined into a single matrix **D_1_**, in order for them to be modelled simultaneously. This can be performed since the source of contrast (i.e., GNRs) has the same spectroscopic properties amongst the two experiments. The background PA properties of the biological tissues are reported in Supplementary Figure S6.
- For the “LIVER” data set, instead, the analysis was performed on a single data set, unfolded in the matrix **D_2_**.

**Figure 3:**
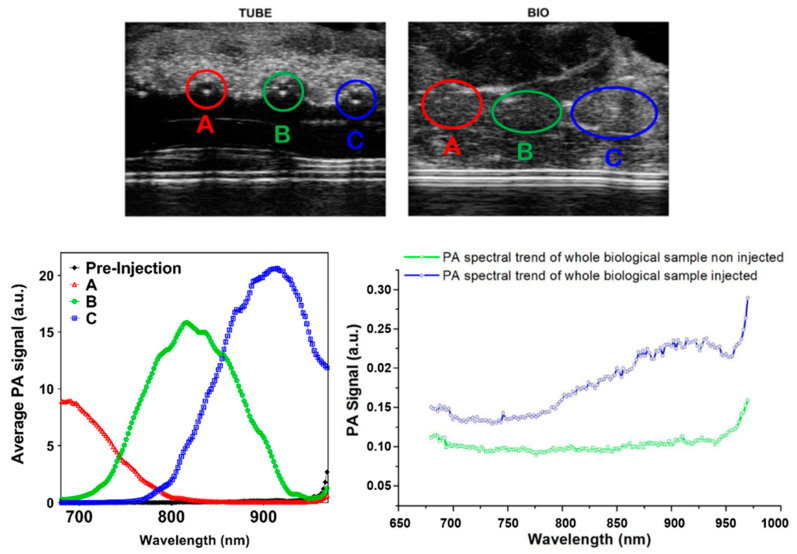
Top: ultrasound images of the analyzed specimens showing the regions in which the three contrast agents (GNRs A, B, and C) were injected. Bottom left: comparison between PA spectra of PEGylated GNRs before and after injection of the three solutions in PE tubes. The PA spectra were obtained by averaging the spectra recorded in 50 × 50 pixel regions. Bottom right: plot of PA spectral trends of the biological sample before and after the injection of the different GNRs. This plot underlines the important role of unmixing algorithm and reveals the specific spectral trend of each one colloidal solution inside the biological tissue.

The MCR-ALS algorithms require an estimate of the spectra of the pure components to proceed with the data compression. Since in the “TUBE” data set the three GNRs solutions were physically separated and distinguishable, their uncontaminated PA spectra were employed for such purpose. This was performed by extracting the average PA spectrum of representative regions (50 × 50 pixels) of the “TUBE” data sets containing the PA response of the separated GNRs solutions and compared to the PA spectrum of the biological tissue recorded prior to injection (Figure 3) revealing strong contrast from the nanostructured materials with partial overlap between different contrast agents but negligible wavelength-nonspecific contribution from the background. These were compared with the VIS-NIR absorbance spectra of GNRs, showing perfect similarity. The input spectra were firstly normalized, then collected in the second input matrix, **x_0_1_**, with a size of 146 × 3.

### 3.3. Multivariate Analysis of “TUBE” and “BIO” Data Sets

**Figure 4:**
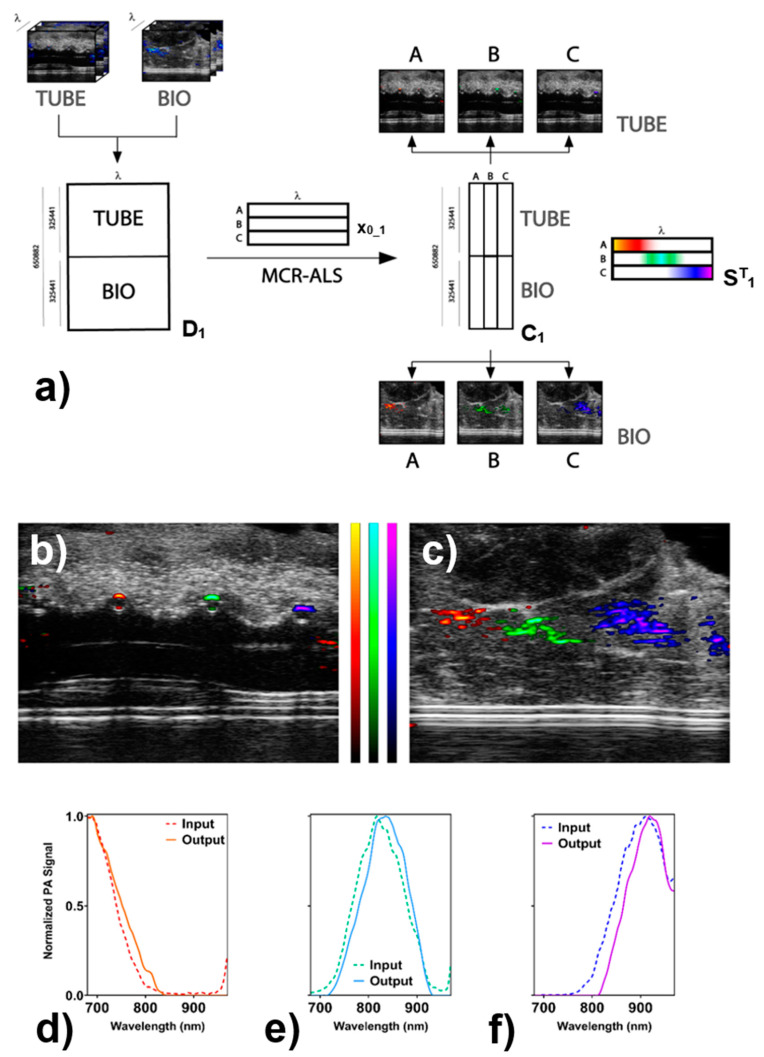
(**a**) Schematic representation of the applied algorithm on “TUBE” and “BIO” data sets. Firstly, the hyperspectral data cubes containing the PA images recorded at different wavelengths are reshaped to bidimensional matrices and merged into the input matrix **D_1_**. Then, by using the pure PA spectra of isolated GNRs A, B, and C in PE tubes (collected in the matrix **x_0_1_**) the MCR-ALS analysis is applied, leading to the obtaining of a reduced matrix **C_1_** containing the distribution profiles of the different GNRs in both data sets and the refined PA spectra of GNRs in the matrix **S^T^**. Matrices sizes are: 503 × 647 × 146 for the hyperspectral data cubes, 650,882 × 146 for matrix **D_1_**, 3 × 146 for **x_0_1_** and **S^T^_1_** and 650,882 × 3 for matrix **C_1_**. (**b**,**c**) Distribution profiles of GNRs obtained by applying the MCR-ALS algorithm to the “TUBE” and “BIO” data sets. Color bars are in arbitrary units and are referred to as GNRs A (red to yellow), GNRs B (green to light blue), and GNRs C (blue to purple). (**d**–**f**) Normalized input and output spectra for the three components are displayed.

MCR-ALS was applied to the multivariate analysis of the sample as reported in Figure 4a. Since “TUBE” and “BIO” data sets were acquired by imaging identical sets of GNRs solutions, they were merged in the same matrix **D_1_** in order to model the two experiments simultaneously. The only applicable constraint of MCR-ALS to PA imaging was found to be the non-negativity of both concentration profiles and spectra. The unimodality constraint could not be applied since it forces the refined spectra to display only one local maximum, and this is misleading in the case of noisy spectra [39,40]. Besides, the closure constraint could not be applied: it acts as a mass balance and implies that the sum of the modelled concentration for all the modelled components is the same everywhere in the image. This is not applicable in multispectral imaging, whereas it finds useful applications in modelling chemical equilibria and reaction kinetics [41]. The intrinsic noise of the experimental technique does not allow for the implementation of unimodality constraints and the closure constraint had to be discarded because during the acquisition time, GNRs have the time to diffuse in and out from the sampled 2D section of the specimen, and a strict mass balance could not be applied. The output of the spectral unmixing was a 650,882 × 3 matrix containing the whole distribution profiles of the three different GNRs in three separate images, **C_1_**. In addition, the algorithm generates the modified spectral profiles for the three-contrast media, **S^T^_1_** such as to satisfy Equation (1) while minimizing the error matrix **E_1_**. The **C_1_** matrix was then separated and reshaped to obtain the pure distribution profiles, which were simultaneously overlapped with the original US traces of the original data sets (Figure 4b,c). Moreover, the output spectra contained in **S^T^_1_** resemble the input ones (Figure 4d–f).

**Figure 5:**
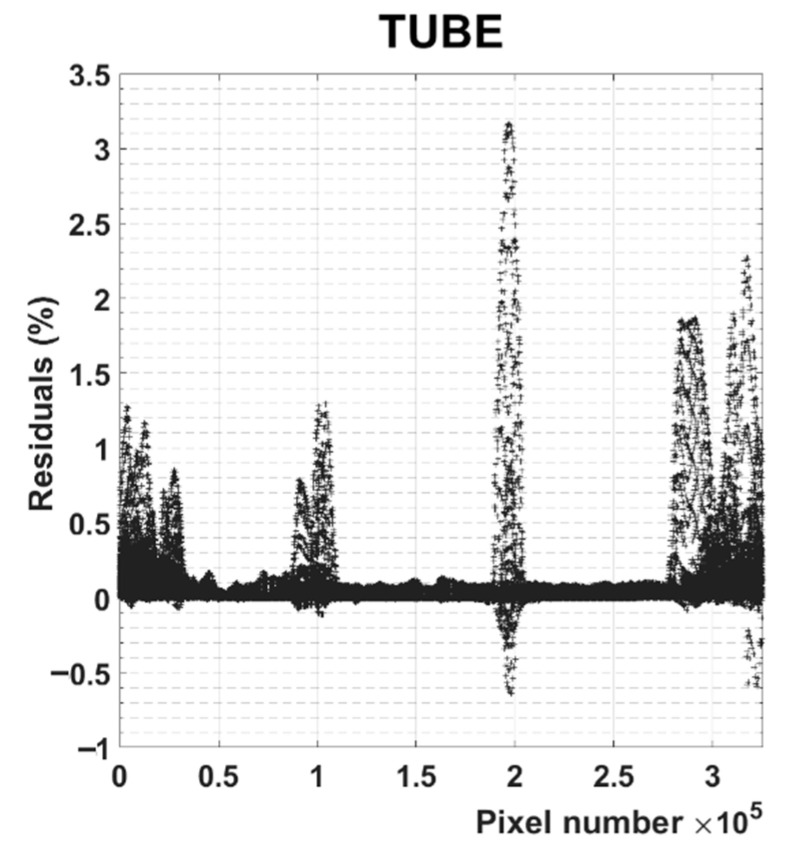

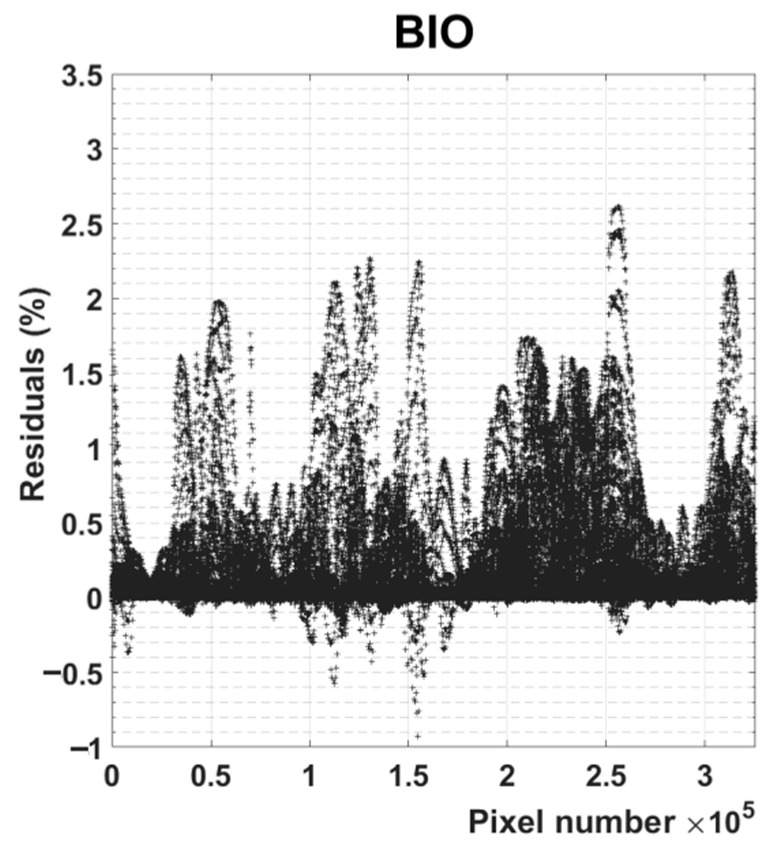
Plot of the maximum residuals for each pixel across all wavelengths, representing the percent of the maximum PA signal recorded for each data set that is not included in the model.

This approach permitted a significant reduction of the size of the data set and was able to compress 650,882 spectra into the combination of three spectra and six distribution images (corresponding to a total reduction of the number of data points from more than 95 million to less than 2 million). Usually, the approach for analyzing multispectral PA imaging involves the isolation from the hypercube of the images corresponding to the maximum absorption wavelengths of the identified chromophores and therefore relating those PA emission maps to the spatial distribution of the corresponding chromophore (as is reported in Supplementary Figure S7). A limitation of this approach is represented by an incorrect balance between intensity and concentration. That is, a recorded low-intensity PA signal could suggest both a low concentration of one chromophore and a much higher concentration of another species that responds poorly to the excitation wavelength in question. For each overlapping region, it is necessary to extract the whole PA spectrum and to assess which is the dominant component. A multivariate approach was applied to process the massive amount of PA data contained in the hypercube (146 images), giving only three images spatially encoded. Here, in the case of overlapping distributions, a strong signal in the unmixed distribution maps corresponds to an intense unresolved signal of the related species. With the presented multivariate approach, the images represent the distributions of the whole spectral features related to the present chromophores, thus allowing for fast and unambiguous discrimination between signals arising from competing molecular species. In the case of overlapping responses, a strong signal in the unmixed distribution maps corresponds to a considerable overall contribution of the related species. Due to the partial overlap between the spectra of GNRs B with GNRs A and C, few pixels show non-zero concentration at the same time for more than a single contrast agent (A and B or B and C). However, by comparing the ratio between the intensity of the modelled signal of the two competing species, it was noticed that in all overlapping spots, one was at least 10 times stronger than the other (Supplementary Figure S8). Therefore, it was chosen to display for each pixel only the contrast species that gave rise to the highest modelled concentration, which led to a distinct separation of the different contributions. Finally, the matrix of residuals (**E_1_**) was evaluated with respect to the corresponding original PA data set, revealing that all the deviations of the modelled data from the raw ones are less than 3% of the maximum amplitude for both data sets (Figure 5 and Supplementary Videos S4 and S5).

### 3.4. Multivariate Analysis of the LIVER Data Set

**Figure 6:**
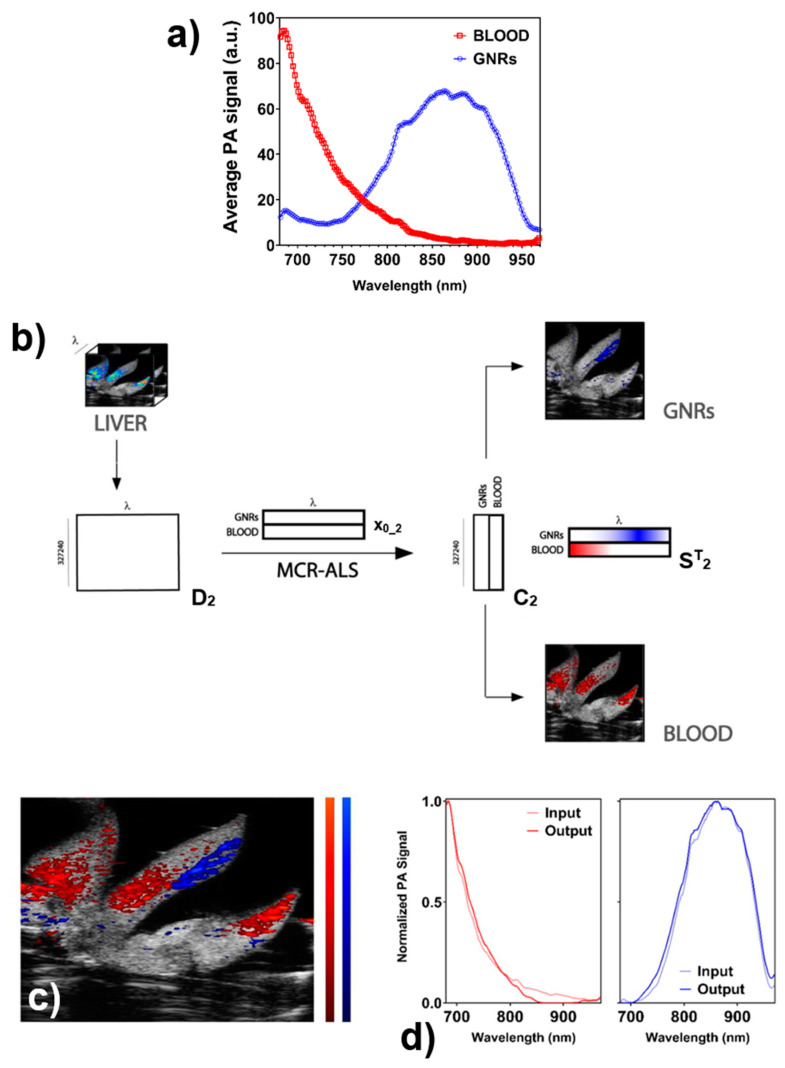
(**a**) Recorded non-normalized PA spectra averaged in a region of 50 × 50 pixels in the lateral lobes (blood, red line) and middle lobe (GNRs, blue line) revealing partial overlap between the spectral profiles and comparable signal intensities. (**b**) Schematic representation of the applied algorithm on the LIVER data set. Firstly, the hyperspectral data cube containing the PA images recorded at different wavelengths is reshaped to the bi-dimensional matrix **D_2_**. Then, by extracting the PA spectra of blood and GNRs from different regions of the image, the matrix **x_0_1_** was built and fed to the MCR-ALS analysis. This led to the obtaining of a reduced matrix **C_2_** containing the separate distribution profiles of GNRs and blood and their refined PA spectra in the matrix **S^T^**. Matrices sizes are: 505 × 648 × 146 for the hyperspectral data cubes, 327,240 × 146 for matrix **D_2_**, 2 × 146 for **x_0_2_** and **S^T^** and 327,240 × 2 for matrix **C_2_**. (**c**) Distribution profiles of GNRs obtained by applying the MCR-ALS algorithm to the LIVER data set. Color bars are in arbitrary units and are referred to GNRs (red) and blood (blue) (**d**). Normalized input and output spectra for the two components displayed.

The PA response of the liver tissue is strongly influenced by the presence of blood; depending on whether it is oxygenated or deoxygenated, hemoglobin displays a distinct spectral profile in the NIR I as reported in Supplementary Figure S9. For setting up the MCR-ALS spectral unmixing of GNRs, no reference material was available for the ex-vivo sample. To extract the input spectra of blood and GNRs, the entire organ was analyzed and the following assumptions were made. Starting from the unmixed PA data (Supplementary Video S3), different profiles were detected in proximal organ regions: the first and third lobes respond mostly in the first part of the NIR I range (680–720 nm, peaked at 684 nm), while the central lobe was shifted to 800–840 nm region, peaked at 862 nm. The difference was sufficiently marked to perform an assignment of the different components, (1) in the middle lobe to GNRs component, while (2) the signal in the lateral lobes to mainly blood. This was confirmed by the ratios between the PA signals of the two regions at the two selected wavelengths: at 684 nm the PA signal of the central lobe was only 1.3% of the signal at 862 nm, and the lateral lobes displayed at 862 nm 5% of the signal recorded at 684 nm. The input spectra for MCR-ALS were collected, averaging a relevant number of PA spectra in those two regions (Supplementary Figure S10). At this level, the algorithm was fed with the new arrays (contained in the matrix **D_2_** with size 327,240 × 145) together with the normalized input spectra (Figure 6a). A simple and fast unmixing of the PA imaging data into the matrix **C_2_**, containing the separated spectral contributions of GNRs and blood (Figure 6b), and the matrix **S^T^_2_** carrying the refined PA spectra of the two components (Figure 6c,d) was obtained. The capacity of separation of the two main components in the analyzed organ provided a precise separation of the distribution profiles of the endogenous and exogenous contribution, with minimal overlapping (Supplementary Figure S11).

**Figure 7:**
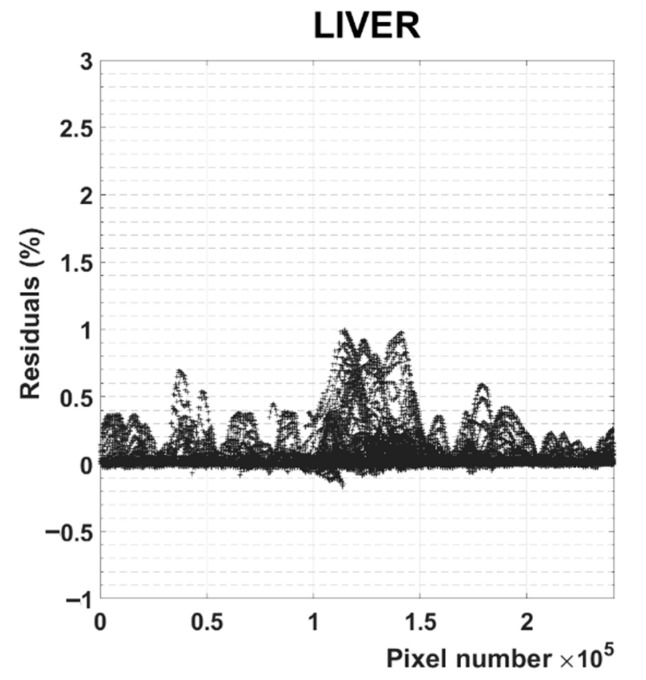
Plot of the maximum residuals for each pixel across all wavelengths, representing the percent of the maximum PA signal recorded for the LIVER data set that is not included in the model.

The multivariate analysis was permitted to compress 327,240 spectra into two spectra and two images that are still able to represent more than 95% of the total variance contained in the initial data set (Supplementary Video S6). In this case, the residuals hardly reach 1% of the maximum PA amplitude recorded for these acquisitions (Figure 7). Additional information about the statistical analysis of the datasets was reported and commented on in the Supplementary Materials. With this approach, good results were obtained without any prior knowledge, i.e., without recording the PA behavior in separate ex-vivo experiments.

### 3.5. Semi-Quantitative Comparison with “Reference Unmixing Tool”

Most PA imaging platforms allow the manipulation of raw data using proprietary software. This is the case for the VEVO Lazr 2100 (Fujifilm Visualsonics, Toronto, ON, Canada), which is supported by the VEVOLAB software that contains a built-in unmixing tool. This data visualization algorithm requires the spectra of the pure components (pre-recorded or available in a recordable repository/library) and spatially separates them, allowing only limited data manipulation from the operator.

**Figure 8:**
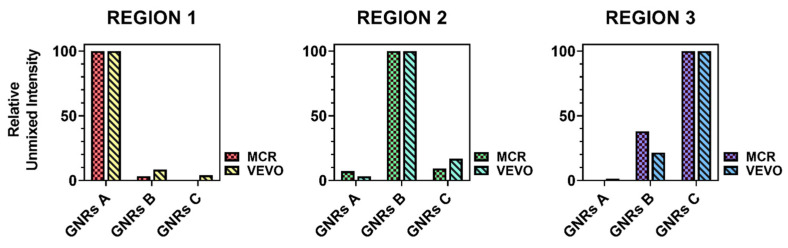
Quantitative comparison of the mismatch in the two unmixing processes for the three responsive components. Relative unmixed intensities were calculated by setting it at 100% for the main species in each distribution map and by averaging the mismatched signals in the same region accordingly.

To evaluate the performances of the presented MCR-ALS approach, with respect to VEVO, the performances of these tools were compared to the “BIO” data set. In total, three regions of interest (ROI of 2.212 mm^2^) were selected around the injection points of three contrast agents (Supplementary Figure S12). Then, separately for each region, the unmixed distribution of each contrast agent was averaged throughout the selected region and plotted in Figure 8 by setting the highest average contribution equal to 100% and calculating the other two accordingly. Ideally, perfect unmixing processing would generate values greater than 0 exclusively for the specific species present in the analyzed region, but, switching to a real case, we expect to find little contributions due to experimental error. The analysis of this mismatch allowed us to assess whether the two unmixing methods deliver comparable results. 

## 4. Conclusions

The most common approach for analyzing multispectral PA imaging involves the isolation from the hypercube of the images corresponding to the maximum absorption wavelengths of the identified components, therefore relating those PA emission maps to the spatial distribution of the corresponding chromophore (Supplementary Figure S5). This approach could lead to an incorrect balance between intensity and concentration. Moreover, the amount of data for images and the time necessary to process them can represent a crucial issue, that needs time and powerful resources in terms of hardware and data storage. In this work, we presented an improved approach based on the MCR-ALS algorithm, leading to good discrimination between signals arising from responsive molecular species. MCR-ALS algorithms were tested for GNRs tuned at different wavelengths to provide a significant compression, reducing the size and shape of multispectral PA raw data. The multivariate analysis was permitted to compress 327,240 spectra into two spectra and two images that are still able to represent more than 95% of the total variance contained in the initial data.

This algorithm also shows efficient results in complex biological tissues with high concentrations of endogenous interfering contrast. The capacity to separate the two main components of the blood well, oxygenated and deoxygenated hemoglobin, represents a really important goal in the data analysis for understanding the real efficiency of the action potential of a contrast agent in a diagnostic or theranostic application. To underline this point, we applied the MCR-ALS algorithm on a mouse liver dataset, in which the tumors were treated with functionalized theranostic agents. Here, the algorithm allowed a precise separation between the spectral contributions of the GNRs, extracted in the lesion, with a minimal overlapping (Supplementary Figures S7 and S9) generated by the blood components.

Future challenges will include the application of the algorithm into a more complex in vivo target, weighting also the spectral contribution generated from the skin by the presence of the melanin, and further reducing spectral interferences got from the blood components.

## Data Availability

The data presented in this study are available on request from the corresponding author. The data are not publicly available due to the fact that the EDIT projects that funded this work is still ongoing.

## Supplementary Materials

The following are available online at https://www.mdpi.com/2079-4991/11/1/142/s1, Figure S1: Schematic representation of a hypercube, Figure S2: Schematic representation of the action of MCR-ALS on hyperspectral data, Figure S3: PA imaging for the “TUBE” data set, Figure S4: PA imaging for the “BIO” data set, Figure S5: Linear correlation between gold concentration and PA signal for GNRs C, Figure S6: Top: Photoacoustic (PA) response of the biological tissue employed for PA experiments (chicken breast as a tissue-mimicking component) before injection of GNRs solutions. Bottom: average PA spectrum of the same tissue after injection of GNRs A, B and C, overlapped with the spectra of the single components (BIO data set). The major contribution in the overall average spectrum (black line) is given by GNRs C (blue line), but contributions from GNRs A (red) and GNRs B (green) is noticeable. Figure S7: Raw PA images for “TUBE” and “BIO” data sets. Figure S8: Unmixed PA images for “TUBE” and “BIO” data sets. Figure S9: PA properties of GNRs-containing polymeric micelles compared to the spectral behaviour of oxy- and deoxy-haemoglobin in blood. Figure S10: Raw PA images for LIVER data set. Figure S11: Unmixed PA images for LIVER data set. Figure S12: Spectral unmixing with the Vevolab software. Table S1: Amounts of reagents employed in the syntheses of GNRs. Table S2: Quantitative description of the in-silico PA tests in terms of PA signal intensity, its standard deviation, coefficient of variation, and signal-to-noise ratio. Table S3: Gaussian fit of the spectral profiles highlighted in Figure S3C. Video S1: Raw PA data from the “TUBE” data set. Video S2: Raw PA data from the “BIO” data set. Video S3: Raw PA data from the LIVER data set. Video S4: Unmodelled data from the “TUBE” data set as residuals of the MCR-ALS analysis. Video S5: Unmodelled data from the “BIO” data set as residuals of the MCR-ALS analysis. Video S6: Unmodelled data from the LIVER data set as residuals of the MCR-ALS analysis.
