# Supplementary material for: An Application of Multivariate Data Analysis to Photoacoustic Imaging for the Spectral Unmixing of Gold Nanorods in Biological Tissues
Source: Nanomaterials (Basel). 2021 Jan 8;11(1):142. doi: 10.3390/nano11010142 (PMC7827716; doi:10.3390/nano11010142)
Supplement: Supplementary file 1 [file nanomaterials-11-00142-s001.zip › nanomaterials-1001648-SI/Supplementary Figures and Tables.docx]

Supplementary Materials

An Application of Multivariate Data Analysis to Photoacoustic Imaging for the Spectral Unmixing of Gold Nanorods in Biological Tissues

Mirko Maturi ^1,^*, Paolo Armanetti ^2^, Luca Menichetti ^2^ and Mauro Comes Franchini ^1^

**THEORY OF MCR-ALS APPROACH**

Photoacoustic imaging implementations often allow for the recording of a photoacoustic signal per each excitation wavelength: if the detector is able to spot a region of the sample of size ($x \times y$) pixels, it would record an image for each available excitation wavelength generating a 3D matrix ($x \times y \times\lambda$) where $\lambda$ is the number of scanned wavelengths. Practically, this matrix is called *hyperspectral cube* (or *hypercube*), and it is composed of images in which for every pixel the entire photoacoustic emission spectrum is recorded (Figure S1); the approach of recording spectra per pixel in 3D matrices, defined as *hyperspectral imaging*, enables the use of powerful statistical tools to discriminate whether the signal is originated from one species or one other, to make quantitative analysis and to reduce the signal-to-noise ratio [1]. Each image represents then regions of the sample in which the corresponding wavelength has a big influence on the total spectrum.


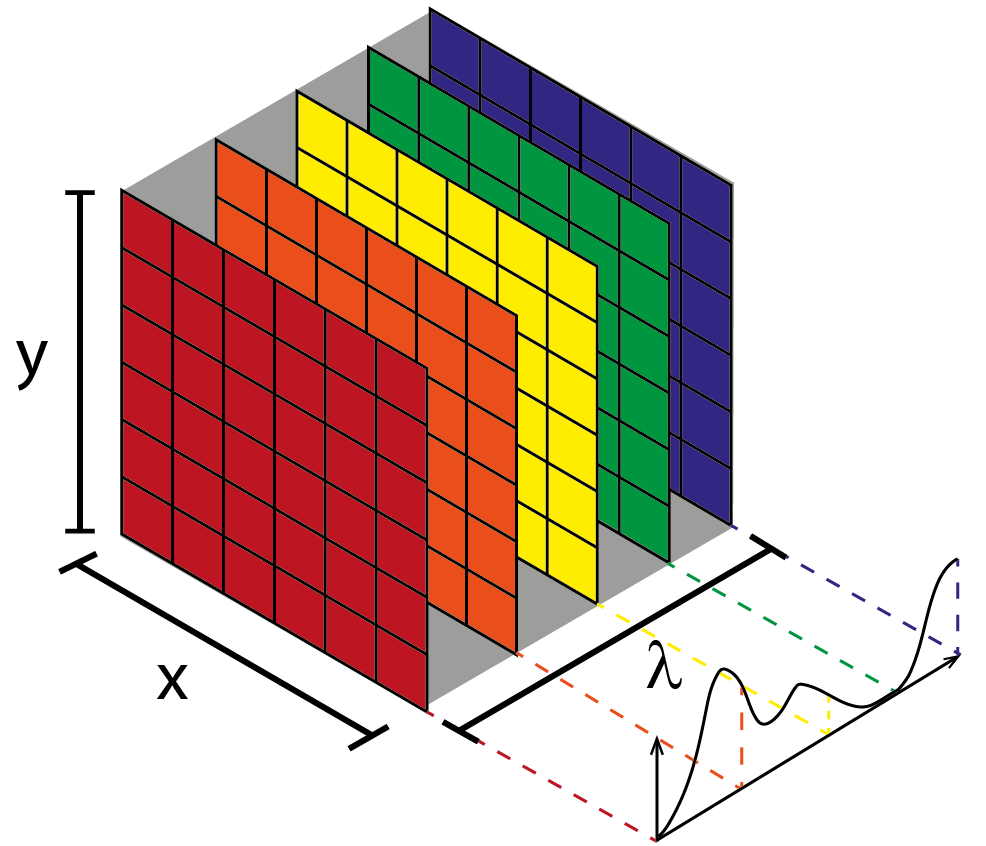


**Figure S1.** Schematic representation of a hypercube. Two dimensions represent the *x*–*y* position of the spotted point (spatial information), the third dimension represent the excitation wavelength (spectral information). Each vertical slice of the cube is an image, while each pixel is a spectrum in the third dimension.

The hypercube ($x \times y \times\lambda$) is firstly unfolded to a 2D data matrix ($\boldsymbol{D}$) of size ($(x \times y) \times\lambda$) in which the rows are spectra of the different pixels. A bilinear model is used to fit the data, producing three matrices, **C** and **S^T^** and **E**, expressed in Equation S1 [2–5].

$\boldsymbol{D=C}\boldsymbol{S}^{\boldsymbol{T}}\boldsymbol{+E}$ (S1)

$\boldsymbol{C}$ is the obtained *concentration matrix* of size ($(x \times y) \times q$) which, once refolded, contains one image for each component $q$ in the spectroscopic system representing the concentration profile of that component; $\boldsymbol{S}^{\boldsymbol{T}}$, the *spectra matrix* has the size of ($q \times\lambda$) and it carries the corresponding spectra of the spotted components while $\boldsymbol{E}$, the *error matrix* which contains the deviation of the model from the experimental data, it is minimized by an iterative least-square algorithm which uses various constraints, to reduce ambiguity (Figures S1 and S2).


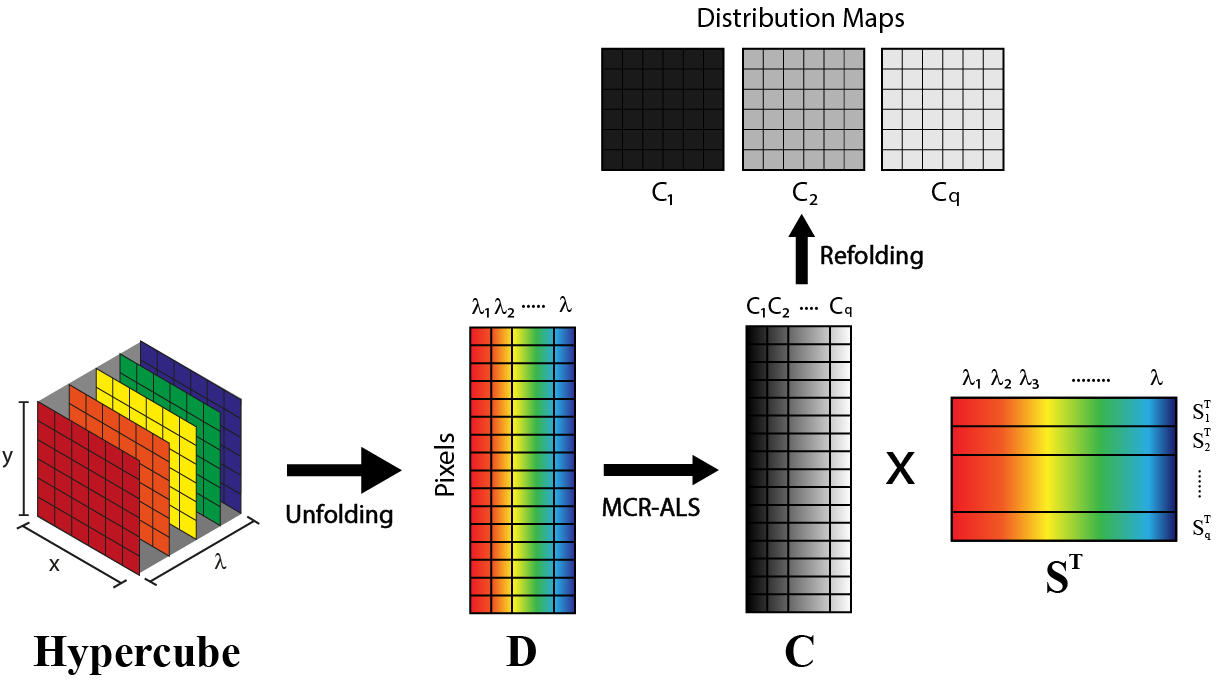


**Figure S2.** Schematic representation of the action of MCR-ALS on hyperspectral data. The hypercube is firstly unfolded, then the algorithm is applied. It generates a matrix $\boldsymbol{C}$ which represents, once refolded, the distribution maps of each of the modelled components, and a matrix$\boldsymbol{S}^{\boldsymbol{T}}$ containing the fitted spectrum for each modelled component.

It should be noticed that Equation S1 is nothing but the multiwavelength expression of the Beer-Lambert law. The algorithm solves iteratively the equation by optimally fitting $\boldsymbol{C}$ and $\boldsymbol{S}^{\boldsymbol{T}}$ by the alternating least-squares algorithm, using initial estimates of the spectral profiles of the mixture components [6]. These inputs can be spectra obtained from reference experiments or can be extracted from the original data set by evolving factor analysis [7]. During the optimization process, several constraints can be applied to model the shapes of the obtained profiles and to limit the number of possible equivalent solutions for the matrix problem. The result is formulated when convergence is achieved in two consecutive iterative cycles, with deviations of the residuals between experimental and ALS data is less than 0.05%.

**Table S1.** Amounts of reagents employed in the syntheses of GNRs A, B and C together with the final gold concentration and yield for the three synthetic batches.

| **Reagent** | **GNRs A** | **GNRs B** | **GNRs C** |
| --- | --- | --- | --- |
| H_2_O | 300 mL | 300 mL | 300 mL |
| CTAB | 10.92 g | 5.40 g | 5.40 g |
| Sodium Oleate | 0 | 0.740 g | 0.740 g |
| AgNO_3_ 0.4 M | 70 μL | 138 μL | 576 μL |
| HAuCl_4_ 0.1 M | 1.5 mL | 1.5 mL | 1.5 mL |
| HCl 37% | 0 | 866 μL | 1.26 mL |
| Ascorbic Acid 78 mM | 2.09 mL | 593 μL | 615 μL |
| Seed Solution | 600 μL | 116 μL | 480 μL |
| Final Volume | 25 mL | 50 mL | 50 mL |
| Final [Au] | 1.46 mM | 1.56 mM | 1.19 mM |
| Reaction Yield | 24.3% | 54% | 50% |

**PA IMAGING AND EXPERIMENTAL SETUP**

**
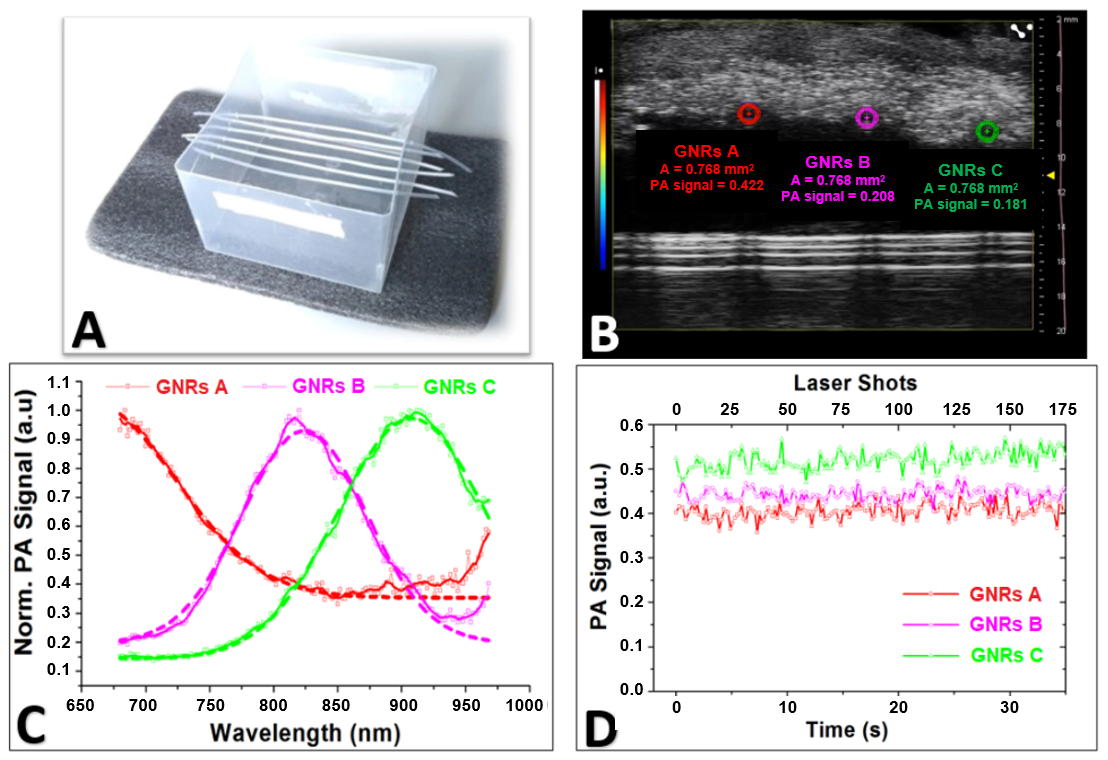
**

**Figure S3.** PA imaging for the TUBE data set. A) Picture of the experimental setup; B) US trace of the analysed system with the localization of the three GNRs solutions; C) Recorded PA spectra in the three confined regions (hollow points), their boxcar average on 5 adjacent points (solid line) and their Gaussian fit (dotted line); D) Recorded PA signal over continuous irradiation at the maximum of PA intensity for each region.

**Table S2.** Quantitative description of the *in silico* PA tests in terms of PA signal intensity, its standard deviation, coefficient of variation and signal-to-noise ratio.

|  | **PA Signal at maximum absorption** | **Standard Deviation** | **Variation Coefficient** | **Signal-to-Noise Ratio (SNR)** |
| --- | --- | --- | --- | --- |
|  | (a.u) | (a.u) | (%) |  |
| **GNRs A** | 0.405 | 0.017 | 4.207 | 24 |
| **GNRs B** | 0.444 | 0.014 | 3.211 | 31 |
| **GNRs C** | 0.523 | 0.021 | 4.030 | 25 |

**Table S3.** Gaussian fit of the spectral profiles highlighted in Figure S3C.

|  | **PA Peak** | **FWHM** | **Normalized Area** |
| --- | --- | --- | --- |
|  | (nm) | (nm) | (a.u.) |
| **GNRs A** | 671 | 134 ± 19 | 94 ± 3 |
| **GNRs B** | 823 | 93 ± 15 | 77 ± 4 |
| **GNRs C** | 908 | 120 ± 5 | 117 ± 7 |

**
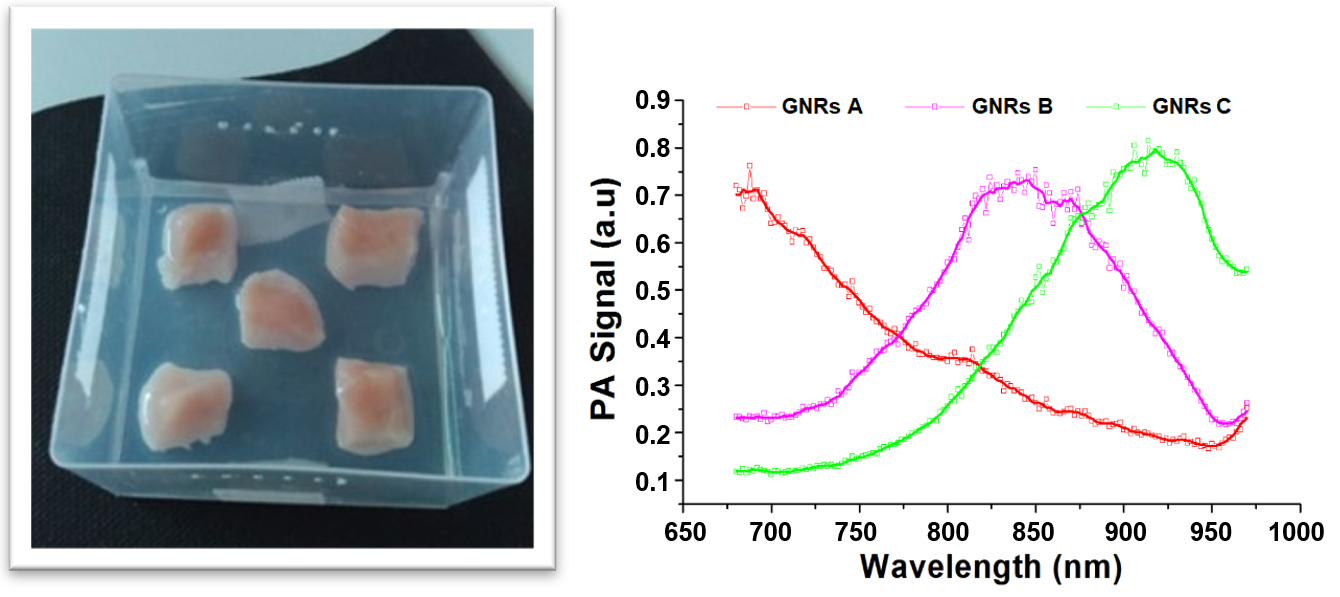
**

**Figure S4.** PA imaging for the BIO data set. Picture of the experimental setup (left) and recorded PA spectra (hollow points) with their boxcar average on 5 adjacent points (solid line) in the three confined regions (right).

**
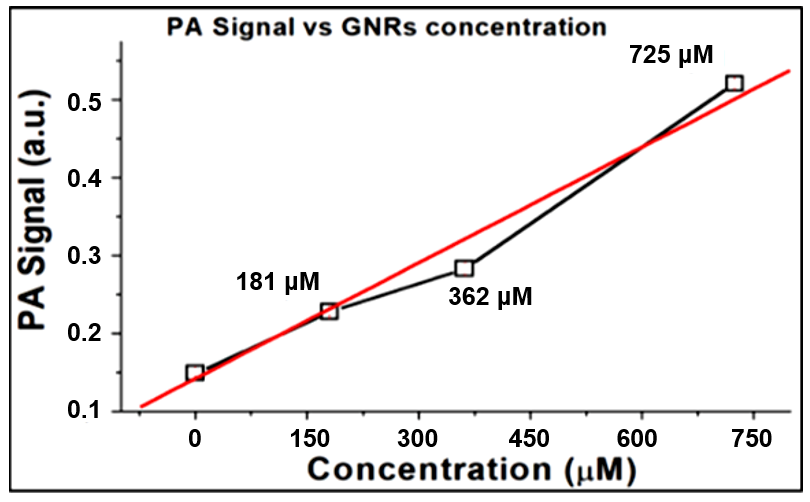
**

**Figure S5.** Linear correlation between gold concentration and PA signal for GNRs C.

**
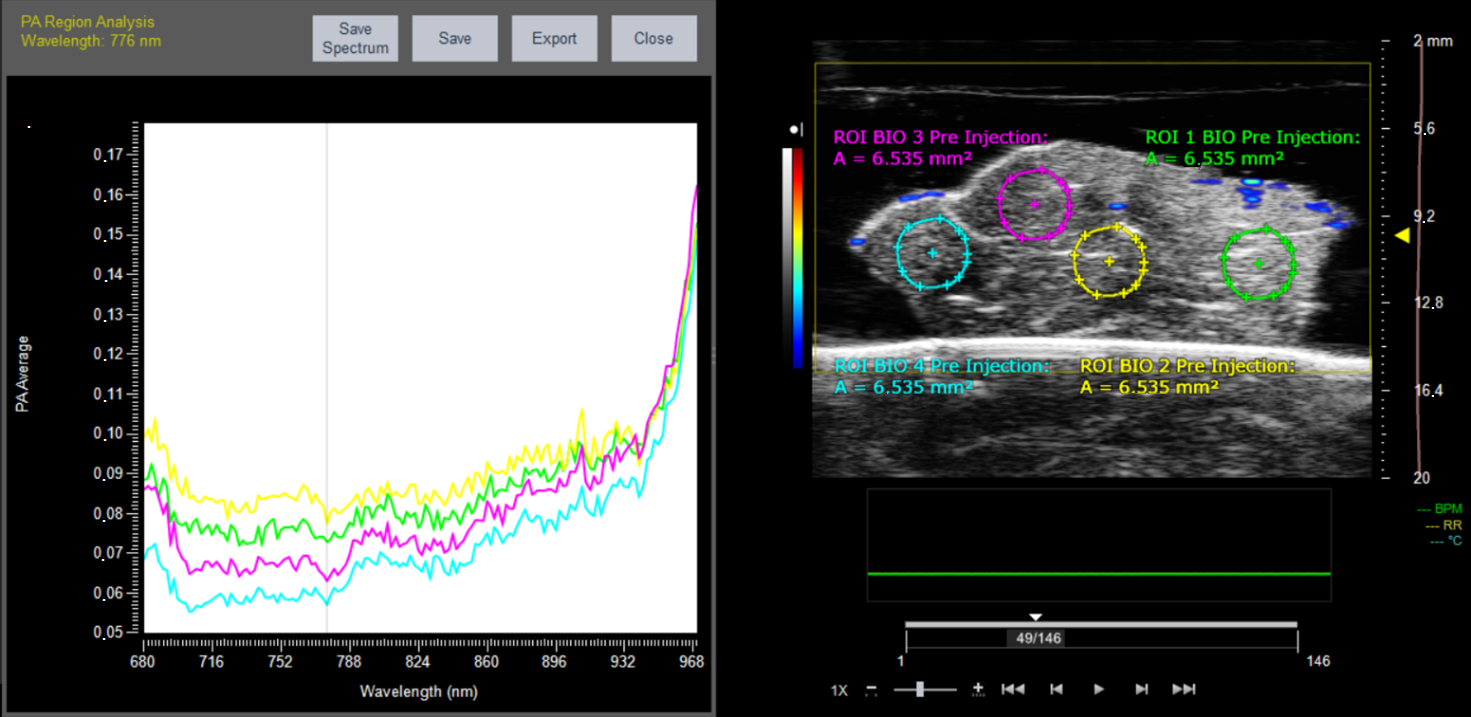
**

**
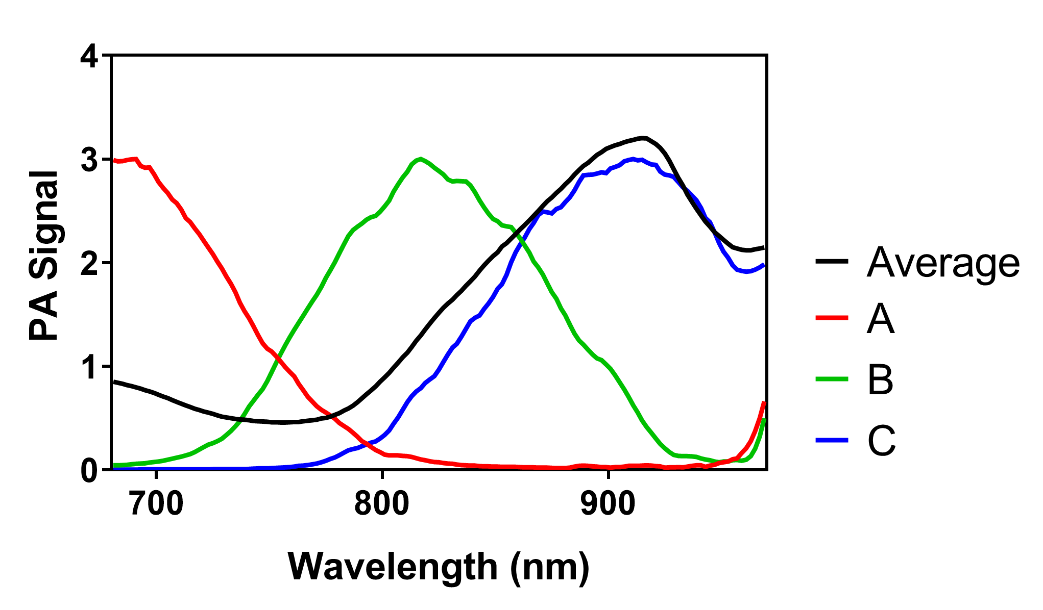
**

**Figure S6.** Top: Photoacoustic (PA) response of the biological tissue employed for PA experiments (chicken breast as tissue-mimicking component) before injection of GNRs solutions**.** Bottom: average PA spectrum of the same tissue after injection of GNRs A, B and C, overlapped with the spectra of the single components (BIO data set). The major contribution in the overall average spectrum (black line) is given by GNRs C (blue line), but contributions from GNRs A (red) and GNRs B (green) is noticeable.

**RAW PA DATA**

*BIO and TUBE data sets*

First of all, noise in the raw PA data have been smoothed applying the Savitsky-Golay filter [8] with 3rd order polynomials on 11 neighboring data points along the dimension representing spectra, then the 3D matrix has been reshaped into a 325,441 × 146 bidimensional matrix in which every row represents the spectrum of one single pixel. Both raw and processed PA data have been overlapped with the co-recorded echographic ultrasound (US) trace setting the transparency conditions at 5% of the maximum, i.e. the PA trace is displayed on the images only for the pixels related to PA emission that is at least 5% of the maximum emission recorded for that specimen (Figure S5).


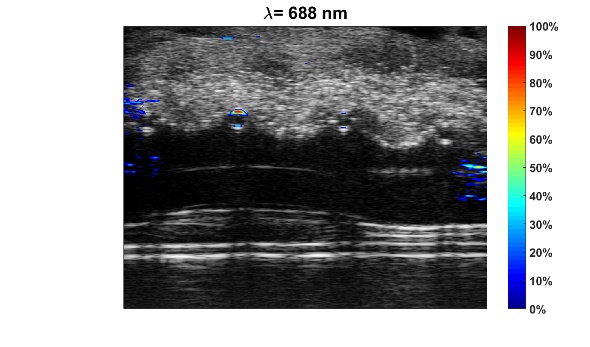

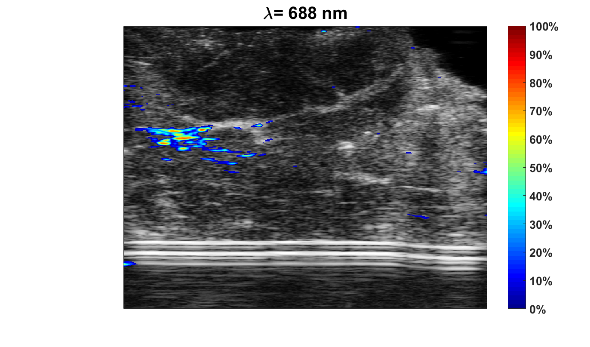

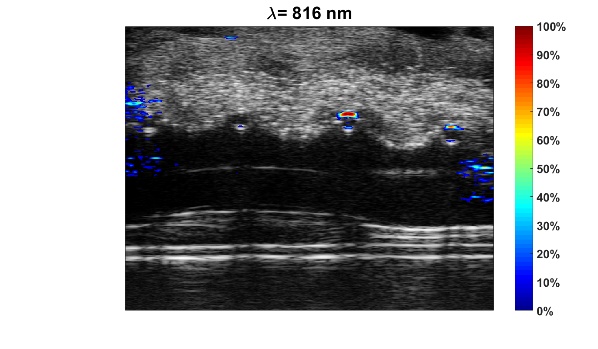

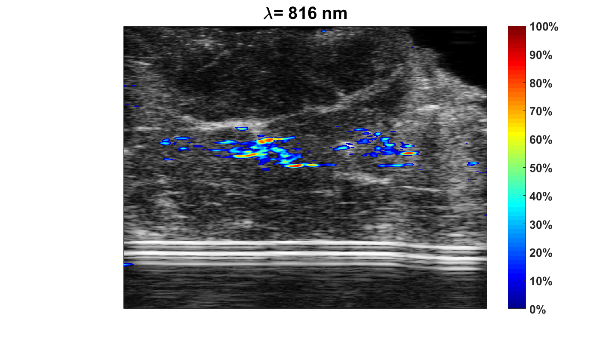

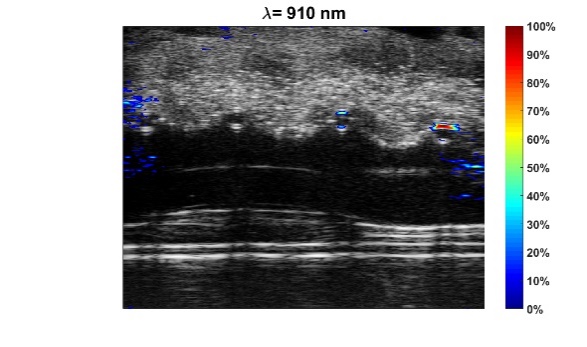

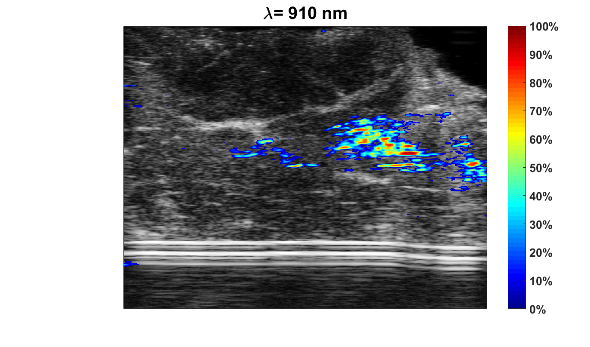


**Figure S7.** Raw PA images (colour scale) overlapped with the co-recorded US trace (grey scale) for both TUBE (left) and BIO (right) data sets at the wavelengths of maximum PA emission for the three explored contrast agents.

**MULTIVARIATE ANALYSIS OUTPUT**

*BIO and TUBE data sets*

By comparison of the images in Figures S5 and S8, it can be easily observed that by applying the MCR-ALS algorithm, ambiguity of the PA contrast is drastically reduced. Even though some overlapping regions are still present after the mathematical analysis, they involve a much smaller number of pixel, and the ratio of the overlapping PA signals is sufficiently reduced.

As before, the modelled trace is displayed on the images only for the pixels related to concentrations that are at least 5% of the maximum modelled concentration for that component.


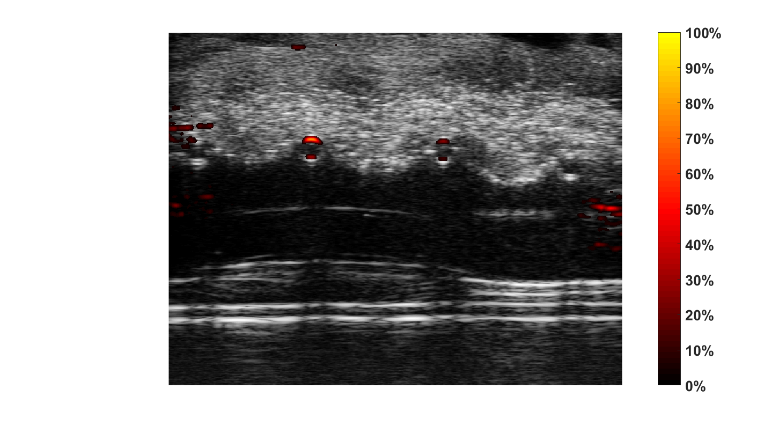

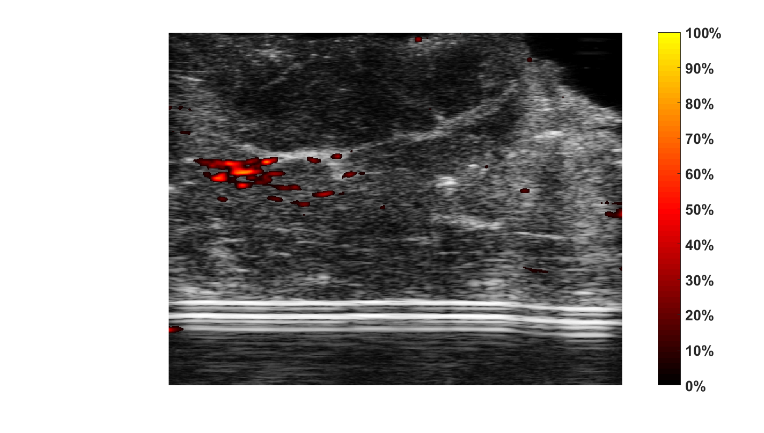

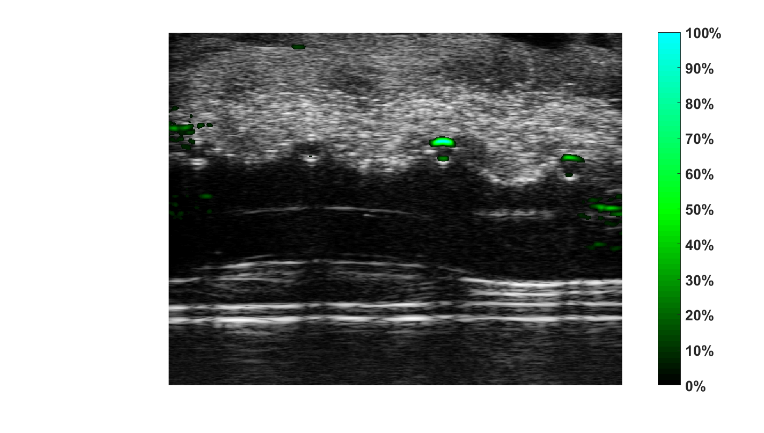

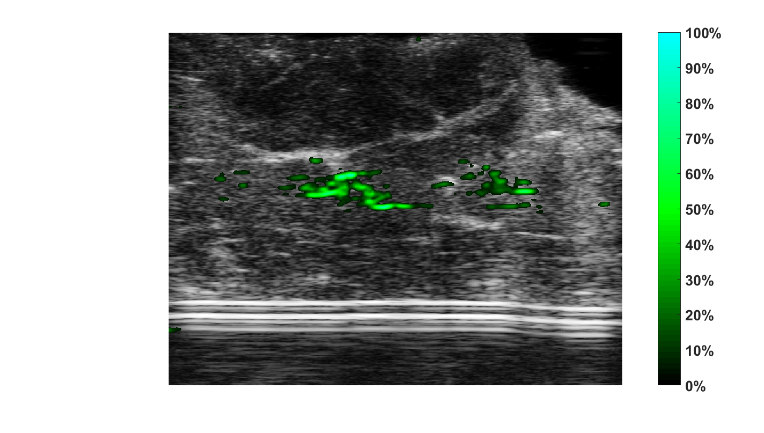

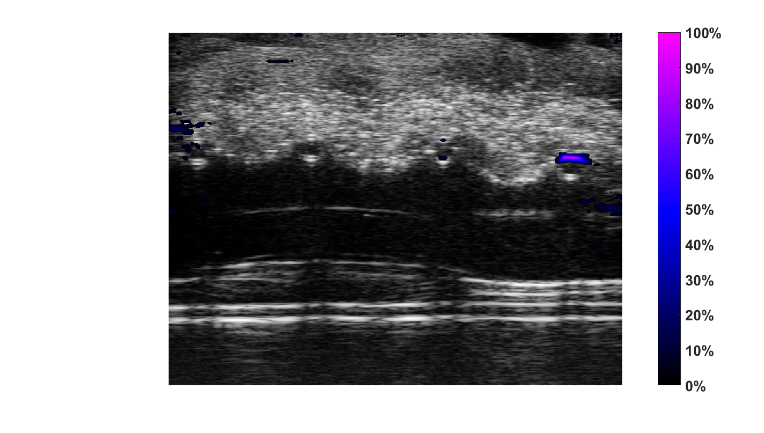

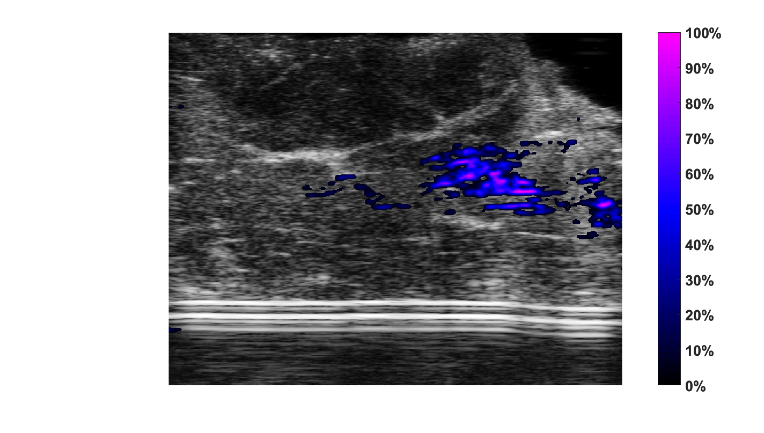


**Figure S8.** Unmixed PA images (colour scale) overlapped with the co-recorded US trace (grey scale) for both TUBE (top) and BIO (bottom) data sets. Colour bars are in arbitrary units and are referred to GNRs A (red to yellow), GNRs B (green to light blue) and GNRs C (blue to purple).

*LIVER data set*

**
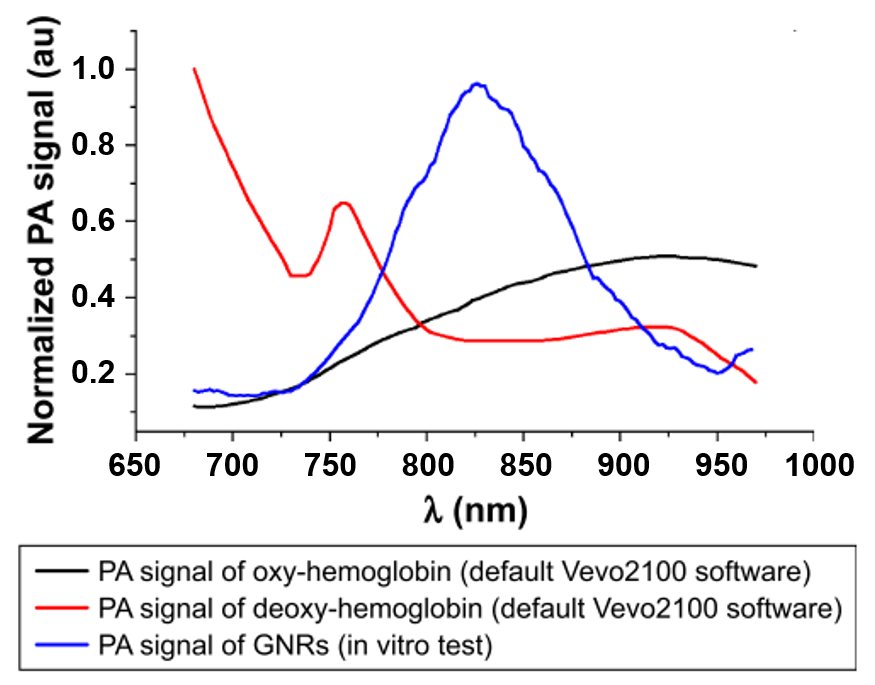
**

**Figure S9.** PA properties of GNRs-containing polymeric micelles compared to the spectral behaviour of oxy- and deoxy-haemoglobin in blood. Reproduced with permission from [9].


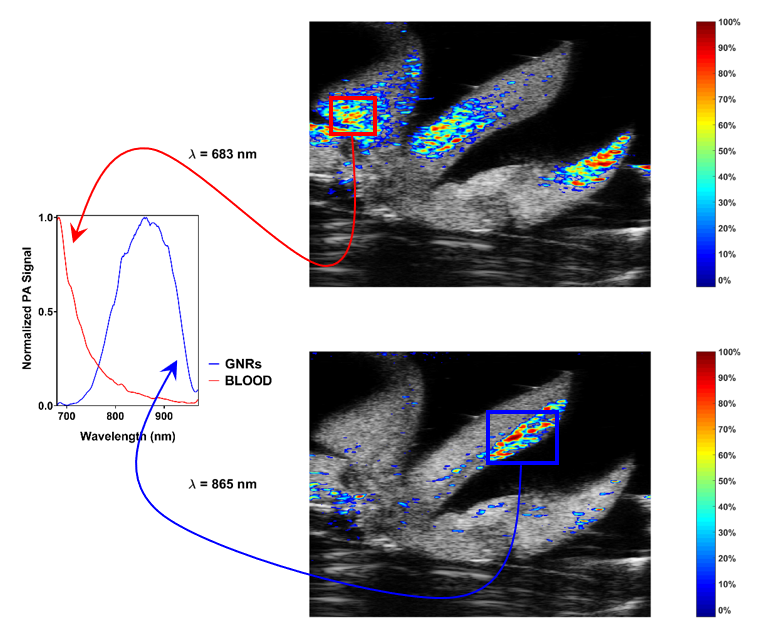


**Figure S10.** Raw PA images (colour scale) overlapped with the co-recorded US trace (grey scale) for the LIVER data set at the wavelengths of maximum PA emission of the two main chromophores. Red and blue boxes identify the regions whose spectra have been averaged to obtain the input matrix **x_0_2_** for the MCR-ALS analysis, as highlighted in the plot at the left.

*LIVER data set*

**
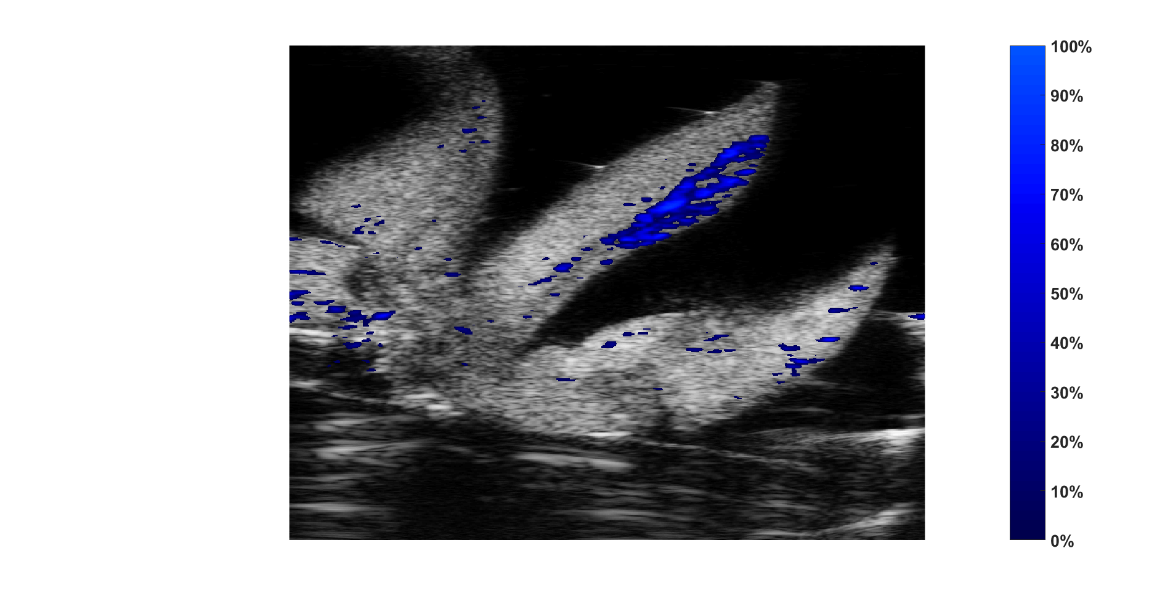
**


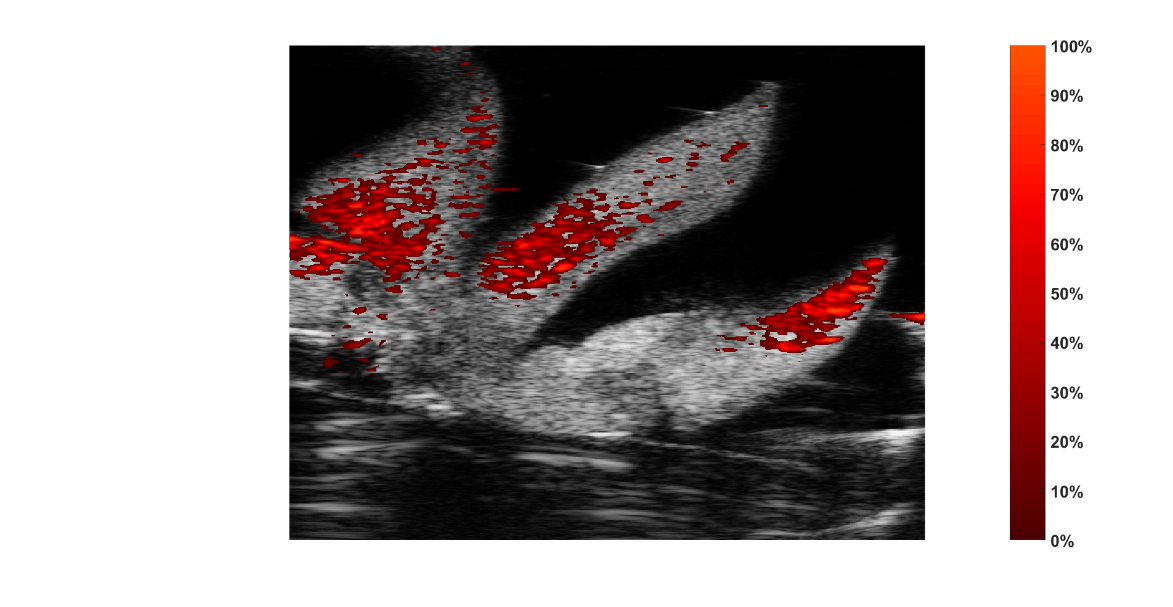


**Figure S11.** Unmixed PA images (colour scale) overlapped with the co-recorded US trace (grey scale) for LIVER data sets. Colour bars are in arbitrary units and are referred to GNRs (blue, top) and blood (red, bottom).

**FIGURES OF MERIT**

*BIO and TUBE data sets*

To fully describe the accuracy of the multivariate method, the MCR-ALS algorithm generates several figures-of-merit, that allows for the evaluation of the goodness of the method. First of all, the percentage of lack of fit at the optimum, which is calculated according to Equation S2, represents the difference between the input data ($\boldsymbol{D}$) and the ones obtained by the model ($\boldsymbol{C}\boldsymbol{S}^{\boldsymbol{T}}$).

$Lack of fit \left( \% \right)=\sqrt{\frac{\sum_{i,j} e_{i,j}^{2}}{\sum_{i,j} d_{i,j}^{2}}}\cdot100$ (S2)

where $d_{i,j}$ indicates the elements of $\boldsymbol{D}$ (i^th^ row and j^th^ column) and $e_{i,j}$ is referred to elements of $\boldsymbol{E}$. In the present case, this value resulted unexpectedly high (21.37%), but as suggested by the developers of the algorithm it is usually related to structured noise or to the presence of unmodellable contributions. This is coherent with the fact that the detection of ultrasound waves often generates random reflection artefacts [10,11].

An additional important parameter for the evaluation of the goodness of the fit is the percentage of variance represented by the model (Equation S3).

$R^{2}=\frac{\sum_{i,j} d_{i,j}^{2}-\sum_{i,j} e_{i,j}^{2}}{\sum_{i,j} d_{i,j}^{2}}$ (S3)

where $d_{i,j}$ and $e_{i,j}$ are the same as above.

Notwithstanding the intrinsic noise and artefacts, the applied model has been able to represent as much as 95.43% of the overall variance of the original data set.

*LIVER data set*

Due to the good spectral and spatial separation of the two chromophores, when applied to this data set the model was able to represent as much as 97% of the original variance, with a lower percentage of lack of fit at the optimum, measured as 17.2%.

**
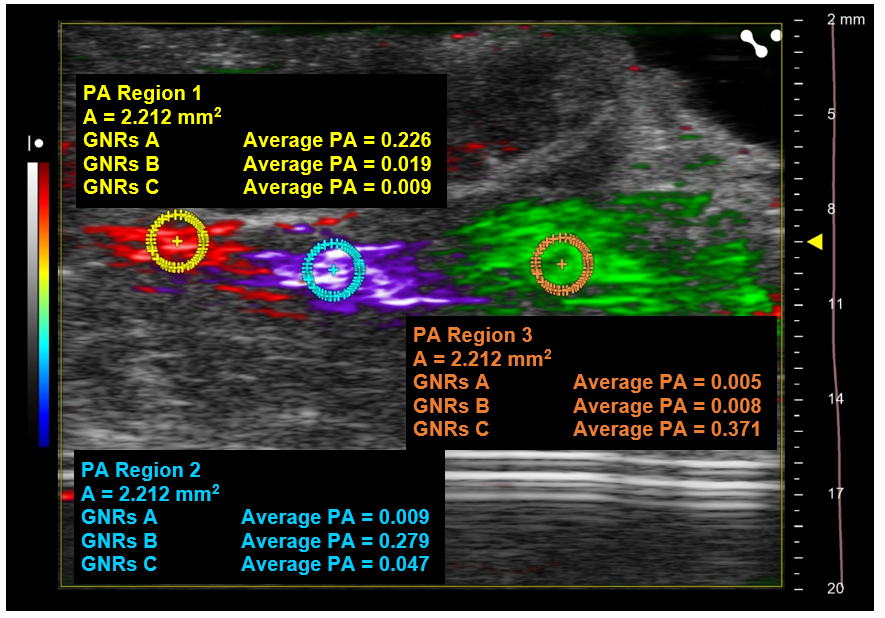
**

**Figure S12.** Spectral unmixing of the VEVOLAB software. Overlapped to the co-recorded US trace, the unmixed distribution maps of GNRs A (red), GNRs B (purple) and GNRs C (green).

**References**

1. Gowen, A.A.; O’Donnell, C.P.; Cullen, P.J.; Downey, G.; Frias, J.M. Hyperspectral imaging—An emerging process analytical tool for food quality and safety control. *Trends Food Sci. Technol.* **2007**, *18*, 590–598, doi:10.1016/j.tifs.2007.06.001.
2. Tauler, R. Multivariate curve resolution applied to second order data. *Chemom. Intell. Lab. Syst.* **1995**, *30*, 133–146, doi:10.1016/0169-7439(95)00047-X.
3. Ruckebusch, C.; Blanchet, L. Multivariate curve resolution: A review of advanced and tailored applications and challenges. *Anal. Chim. Acta* **2013**, *765*, 28–36, doi:10.1016/j.aca.2012.12.028.
4. De Juan, A.; Tauler, R. Multivariate Curve Resolution (MCR) from 2000: Progress in concepts and applications. *Crit. Rev. Anal. Chem.* **2006**, *36*, 163–176.
5. de Juan, A.; Tauler, R. Chemometrics applied to unravel multicomponent processes and mixtures: Revisiting latest trends in multivariate resolution. *Anal. Chim. Acta* **2003**, *500*, 195–210, doi:10.1016/S0003-2670(03)00724-4.
6. Comon, P.; Luciani, X.; de Almeida, A.L.F. Tensor decompositions, alternating least squares and other tales. *J. Chemom.* **2009**, *23*, 393–405, doi:10.1002/cem.1236.
7. Maeder, M. Evolving Factor Analysis for the Resolution of Overlapping Chromatographic Peaks. *Anal. Chem.* **1987**, *59*, 527–530, doi:10.1021/ac00130a035.
8. Savitzky, A.; Golay, M.J.E. Smoothing and Differentiation of Data by Simplified Least Squares Procedures. *Anal. Chem.* **1964**, *36*, 1627–1639, doi:10.1021/ac60214a047.
9. Locatelli, E.; Li, Y.; Monaco, I.; Guo, W.; Maturi, M.; Menichetti, L.; Armanetti, P.; Martin, R.; Comes Franchini, M. A novel theranostic gold nanorods- and Adriamycin-loaded micelle for EpCAM targeting, laser ablation, and photoacoustic imaging of cancer stem cells in hepatocellular carcinoma. *Int. J. Nanomed.* **2019**, *14*, 1877–1892, doi:10.2147/IJN.S197265.
10. Nguyen, H.N.Y.; Hussain, A.; Steenbergen, W. Reflection artifact identification in photoacoustic imaging using multi-wavelength excitation. *Biomed. Opt. Express* **2018**, *9*, 4613–4630, doi:10.1364/BOE.9.004613.
11. Paltauf, G.; Nuster, R. Artifact removal in photoacoustic section imaging by combining an integrating cylindrical detector with model-based reconstruction. *J. Biomed. Opt.* **2014**, *19*, 26014, doi:10.1117/1.JBO.19.2.026014.
