# Supplementary material for: An Application of Multivariate Data Analysis to Photoacoustic Imaging for the Spectral Unmixing of Gold Nanorods in Biological Tissues
Source: Nanomaterials (Basel). 2021 Jan 8;11(1):142. doi: 10.3390/nano11010142 (PMC7827716; doi:10.3390/nano11010142)
Supplement: Supplementary file 1 [file nanomaterials-11-00142-s001.zip › nanomaterials-1001648-SI/Supplementary Videos.pptx]

## Slide 1
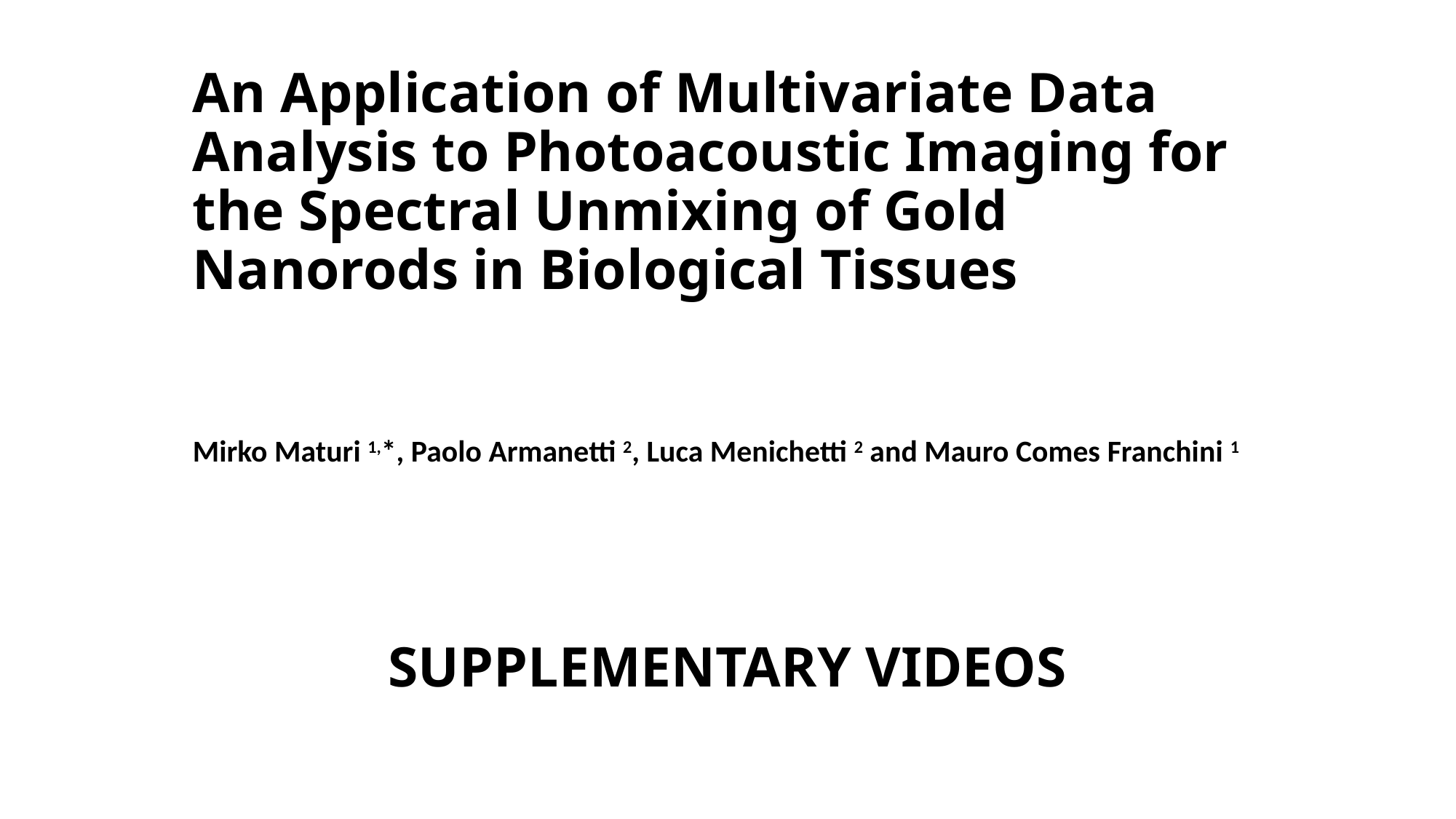

# An Application of Multivariate Data Analysis to Photoacoustic Imaging for the Spectral Unmixing of Gold Nanorods in Biological Tissues
SUPPLEMENTARY VIDEOS
Mirko Maturi 1,*, Paolo Armanetti 2, Luca Menichetti 2 and Mauro Comes Franchini 1

## Slide 2
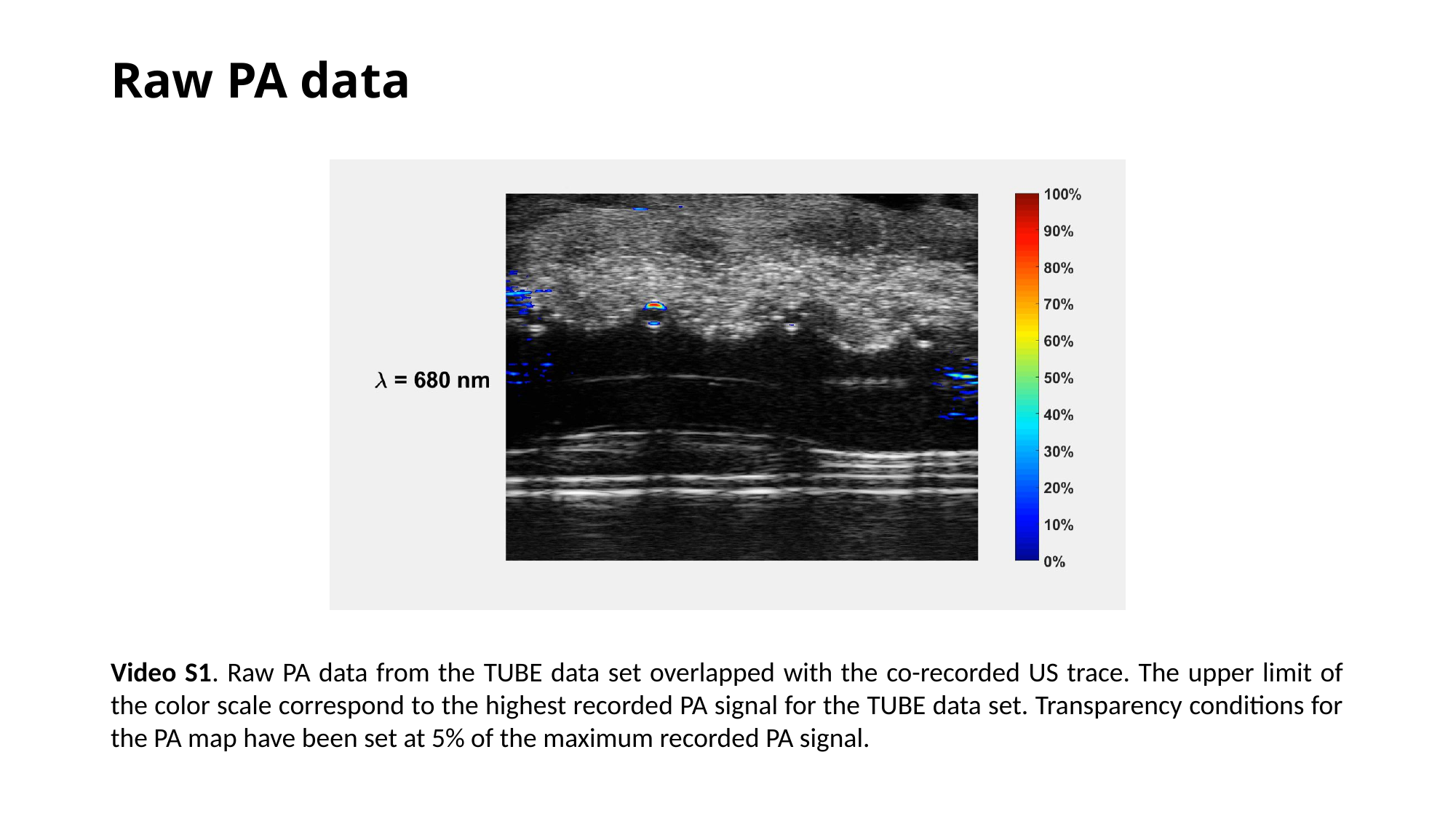

# Raw PA data
Video S1. Raw PA data from the TUBE data set overlapped with the co-recorded US trace. The upper limit of the color scale correspond to the highest recorded PA signal for the TUBE data set. Transparency conditions for the PA map have been set at 5% of the maximum recorded PA signal.

## Slide 3
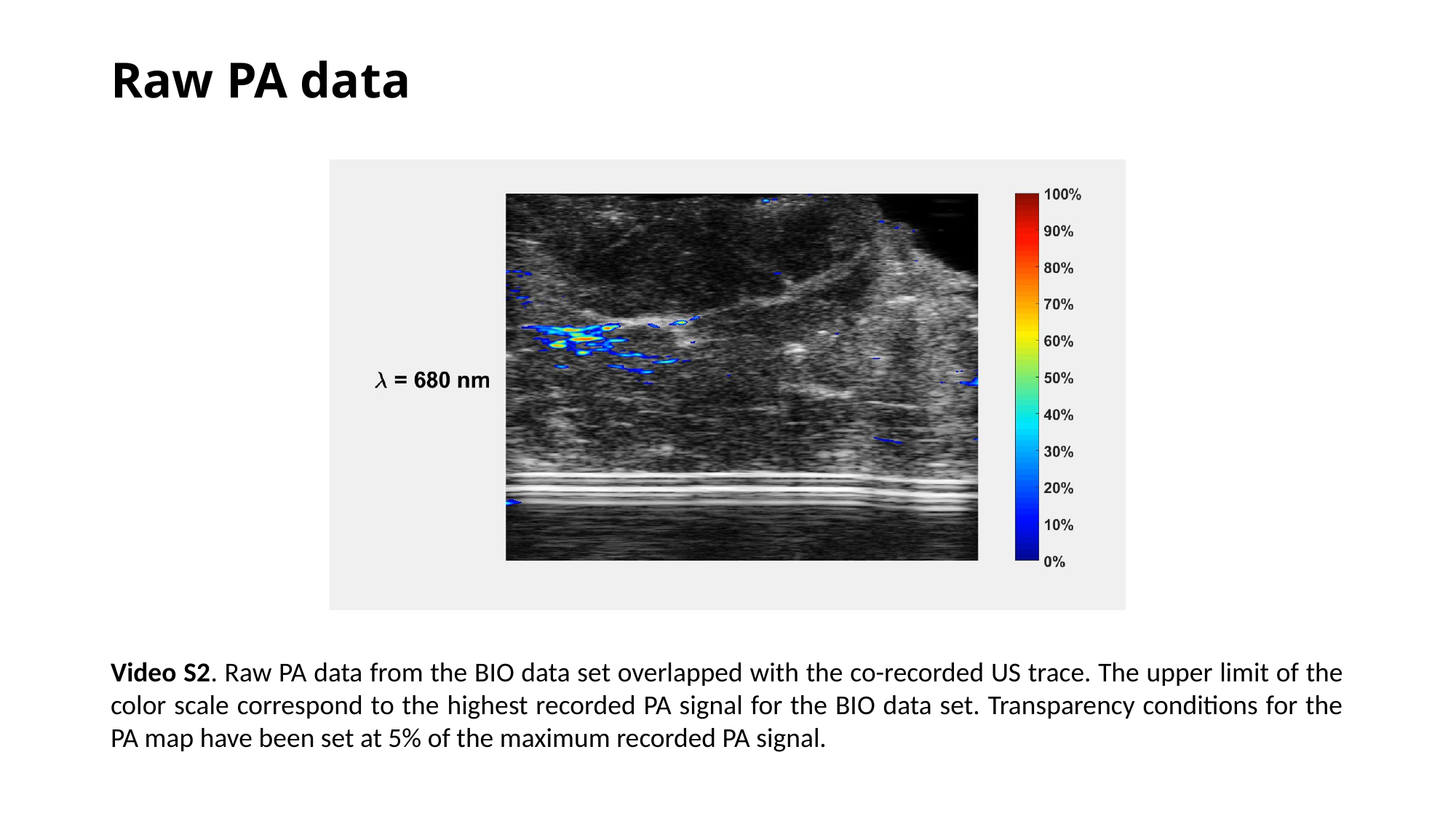

# Raw PA data
Video S2. Raw PA data from the BIO data set overlapped with the co-recorded US trace. The upper limit of the color scale correspond to the highest recorded PA signal for the BIO data set. Transparency conditions for the PA map have been set at 5% of the maximum recorded PA signal.

## Slide 4
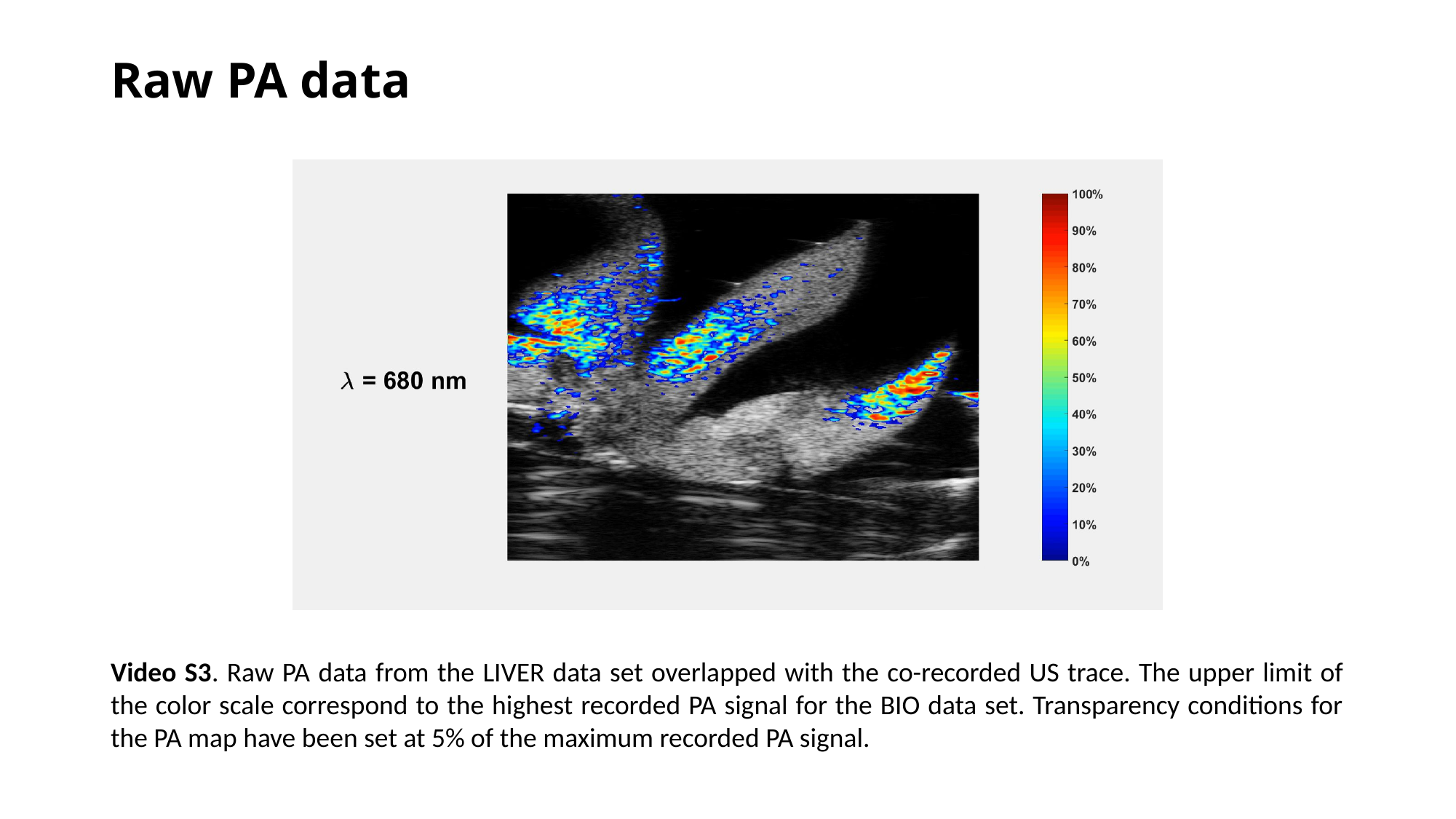

# Raw PA data
Video S3. Raw PA data from the LIVER data set overlapped with the co-recorded US trace. The upper limit of the color scale correspond to the highest recorded PA signal for the BIO data set. Transparency conditions for the PA map have been set at 5% of the maximum recorded PA signal.

## Slide 5
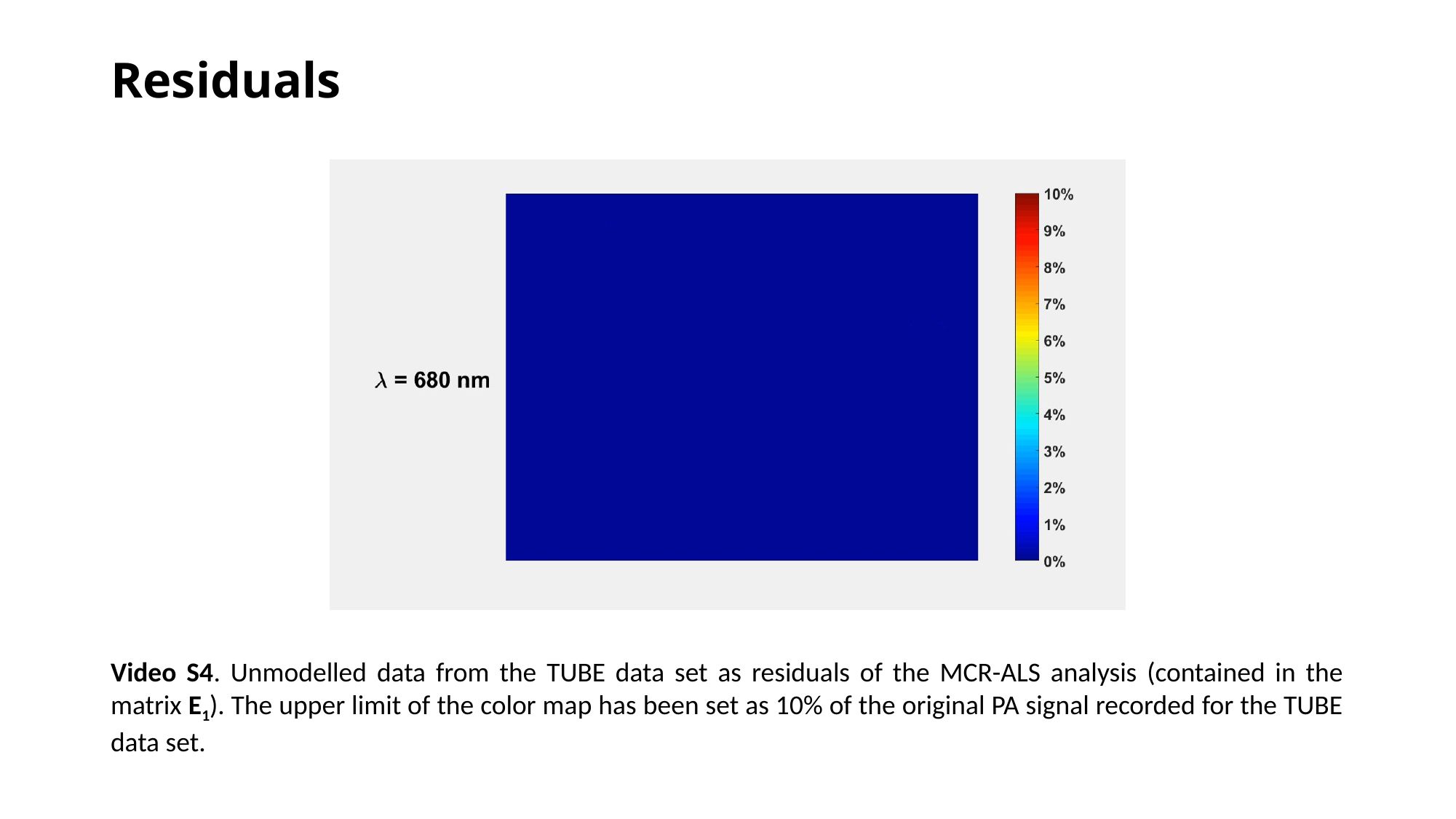

# Residuals
Video S4. Unmodelled data from the TUBE data set as residuals of the MCR-ALS analysis (contained in the matrix E1). The upper limit of the color map has been set as 10% of the original PA signal recorded for the TUBE data set.

## Slide 6
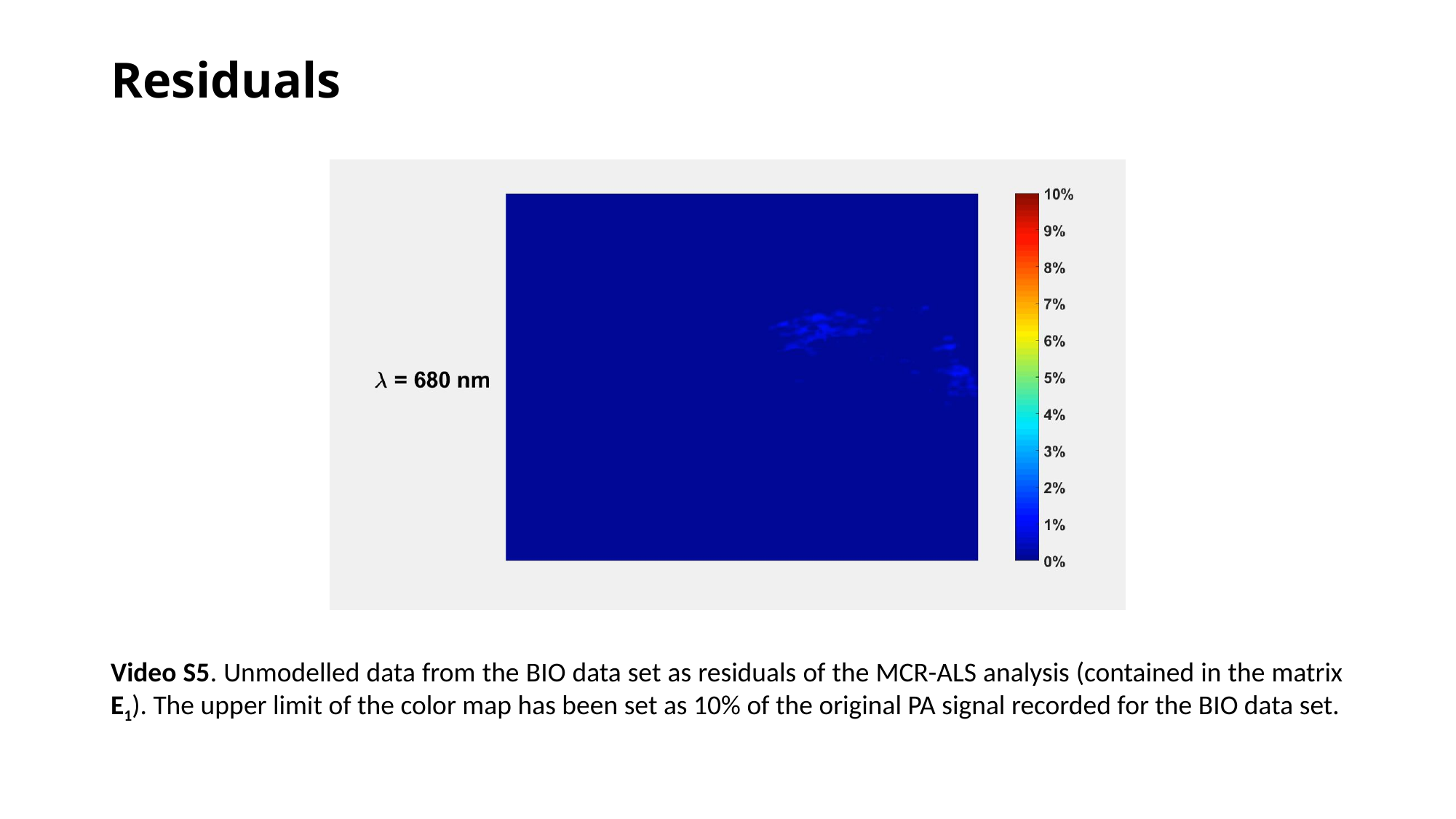

# Residuals
Video S5. Unmodelled data from the BIO data set as residuals of the MCR-ALS analysis (contained in the matrix E1). The upper limit of the color map has been set as 10% of the original PA signal recorded for the BIO data set.

## Slide 7
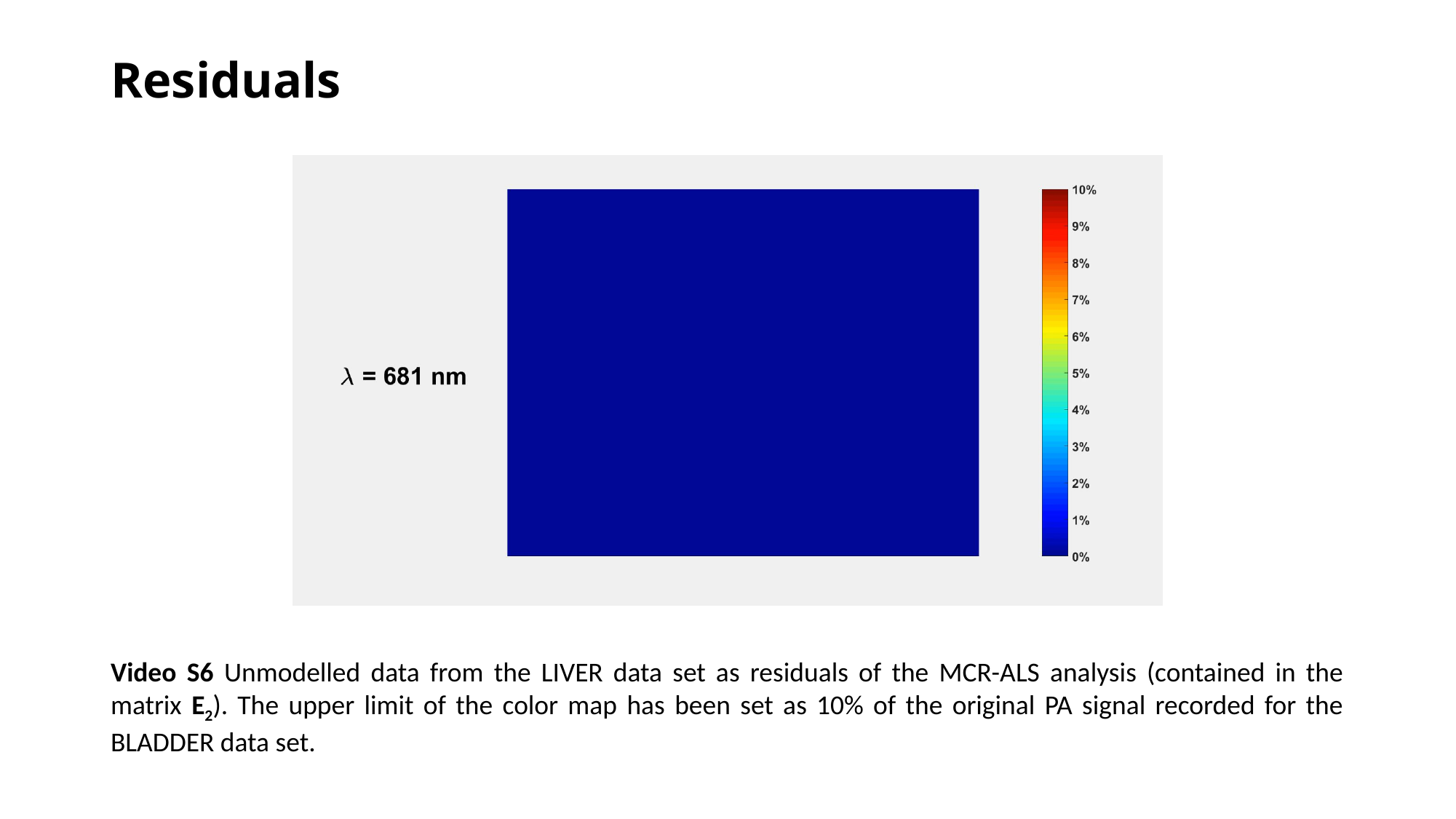

# Residuals
Video S6 Unmodelled data from the LIVER data set as residuals of the MCR-ALS analysis (contained in the matrix E2). The upper limit of the color map has been set as 10% of the original PA signal recorded for the BLADDER data set.
